# The Diagnostic Process of Spinal Post-traumatic Deformity: An Expert Survey of 7 Cases, Consensus on Clinical Relevance Does Exist

**DOI:** 10.1097/BSD.0000000000001478

**Published:** 2023-06-26

**Authors:** Erin E.A. De Gendt, Lorin M. Benneker, Andrei F. Joaquim, Mohammad El-Sharkawi, Gaurav R. Dhakal, Frank Kandziora, Jin Tee, Richard J. Bransford, Emiliano N. Vialle, Alex R. Vaccaro, Eugen C. Popescu, Rishi M. Kanna, David W. Polly, Klaus J. Schnake, Pedro Berjano, Sergey Ryabykh, Marko Neva, Claudio Lamartina, Dominique A. Rothenfluh, Stephan J. Lewis, Sander P.J. Muijs, F. Cumhur Oner

**Affiliations:** * Department of Orthopedics, University Medical Center Utrecht, Utrecht, The Netherlands; † Orthopedic Department, Sonnenhofspital, Bern, Switzerland; ‡ Department of Neurosurgery, State University of Campinas, Campinas, Brazil; § Department of Orthopaedic and Trauma Surgery, Assiut University Medical School, Assiut, Egypt; ∥ National Trauma Center, Bir Hospital, National Academy of Medical Sciences, Kathmandu, Nepal; ¶ Center for Spinal Surgery and Neurotraumatology, BG Unfallklinik Frankfurt am Main gGmbH, Frankfurt, Germany; # Departement of Neurosurgery, The Alfred Hospital, Melbourne, Australia; ** Department of Orthopaedics, University of Washington, Seattle, WA; †† Department of Orthopaedics, Cajuru Hospital, Catholic University of Paraná, Curitiba, Brazil; ‡‡ Department of Orthopaedic Surgery, Rothman Institute, Thomas Jefferson University, Philadelphia, PA; §§ “Prof. Dr. N. Oblu” Emergency Hospital, Iasi, Romania; ∥∥ Department of Orthopaedic and Spine Surgery, Ganga Hospital, Coimbatore, India; ¶¶ Department of Orthopaedic Surgery, University of Minnesota, Minneapolis, MN; ## Center for Spinal and Scoliosis Therapy, Malteser Waldkrankenhaus St. Marien, Erlangen; *** IRRCS Istituto Ortopedico Galeazzi, Milano; ††† National Ilizarov Medical Research Center for Traumatology and Ortopaedics, Russia; ‡‡‡ Theater and Spine Surgery, Tampere University Hospital, Finland Unit, Tampere, Finland; §§§ Nuffield Orthopaedic Centre, Oxford University Hospitals NHS Trust, Oxford, England; ∥∥∥ Toronto Western Hospital, University Health Network, Toronto, ON, Canada

**Keywords:** spinal post-traumatic deformity, consensus, spine trauma, diagnosis

## Abstract

**Study Design:**

Survey of cases.

**Objective:**

To evaluate the opinion of experts in the diagnostic process of clinically relevant Spinal Post-traumatic Deformity (SPTD).

**Summary of Background Data:**

SPTD is a potential complication of spine trauma that can cause decreased function and quality of life impairment. The question of when SPTD becomes clinically relevant is yet to be resolved.

**Methods:**

The survey of 7 cases was sent to 31 experts. The case presentation was medical history, diagnostic assessment, evaluation of diagnostic assessment, diagnosis, and treatment options. Means, ranges, percentages of participants, and descriptive statistics were calculated.

**Results:**

Seventeen spinal surgeons reviewed the presented cases. The items’ fracture type and complaints were rated by the participants as more important, but no agreement existed on the items of medical history. In patients with possible SPTD in the cervical spine (C) area, participants requested a conventional radiograph (CR) (76%–83%), a flexion/extension CR (61%–71%), a computed tomography (CT)-scan (76%–89%), and a magnetic resonance (MR)-scan (89%–94%). In thoracolumbar spine (ThL) cases, full spine CR (89%–100%), CT scan (72%–94%), and MR scan (65%–94%) were requested most often. There was a consensus on 5 out of 7 cases with clinically relevant SPTD (82%–100%). When consensus existed on the diagnosis of SPTD, there was a consensus on the case being compensated or decompensated and being symptomatic or asymptomatic.

**Conclusions:**

There was strong agreement in 5 out of 7 cases on the presence of the diagnosis of clinically relevant SPTD. Among spine experts, there is a strong consensus to use CT scan and MR scan, a cervical CR for C-cases, and a full spine CR for ThL-cases. The lack of agreement on items of the medical history suggests that a Delphi study can help us reach a consensus on the essential items of clinically relevant SPTD.

**Level of Evidence:**

Level V

Spinal post-traumatic deformity (SPTD) is a potential complication after a spine trauma. Patients with a deformity after spine trauma can suffer from a neurological deficit, functional disability, and more commonly back or neck pain.^1–5^

Although all nontrivial spinal injuries result in some deformity, it is not always clear when a patient suffers from a SPTD? In other words, when does a post-traumatic deformity become clinically relevant?

Recently, the AO Spine Knowledge Forum Trauma (KF Trauma) set up a project in search of a consensus definition of clinically relevant SPTD. To construct a consensus definition, a Delphi study will be held among the AO Spine community. Already 2 studies were completed to gather information for the adjusted Delphi study: a systematic review of the literature and a survey of experts.^6,7^ The systematic review showed that there is no clear consensus in the literature about the diagnosis of SPTD, but identified the different domains of the definition or description of SPTD: Radiologic parameters, patient factors, Patient-reported Outcome Measurements, and indication for surgical intervention.^6^

The survey showed that there was some consensus among the 15 spine experts who completed the study. Consensus was reached that pain is an essential criterion for the definition of SPTD. The radiologic assessment deemed necessary for diagnosis and treatment was a full spine conventional radiograph (CR). The only risk factor with a substantial agreement was the factor “missed B-type injury.” There was no agreement on other risk factors leading to clinically relevant SPTD. Concerning the management, all participants agreed that an asymptomatic patient should not undergo surgical treatment and that neurological deficit is an absolute surgical indication.^7^

However, both studies did not help in understanding the thought process behind the diagnosis of SPTD. SPTD is still an ill-defined clinical problem of patients with a deformity and/or persistent complaints after spine trauma. To evaluate this thought process of international spine experts, we constructed a case survey with fully anonymized data of patients who had visited our outpatient clinic in the past.

The aim of this survey is to evaluate the opinion of spine trauma and deformity experts in the diagnostic process of clinically relevant SPTD. Specifically with the questions of whether consensus exists on the more important parameters of the medical history/physical examination, the necessary radiologic assessment, and the more important measurements on radiologic assessments. Also, whether there is consensus on the diagnosis of SPTD and if this is considered clinically relevant, and whether there is consensus on the preferred treatment. This case survey will aid in the development of a Delphi study to create a consensus definition of “clinically relevant SPTD.”

## METHODS

### Study Design and Recruitment of Experts

The experts (orthopedic surgeons or neurosurgeons with research experience) were recruited through the AO Spine Knowledge Forum (KF) Trauma and the KF Deformity. The AO Spine KFs are expert-driven working groups generating knowledge in different spine pathologies. They are tasked to assess the best evidence for current practices and formulate clinical studies to advance their field of spine expertise. The development of the case discussion study was based on KF members’ discussion and the results from the previous systematic review and exploratory survey.^6,7^

### Case Survey Development

Each case was presented as if the patient presented him or herself at the outpatient spine clinic. The case was deemed eligible if the patient suffered from a spine trauma at least 3 months previously. Seven different cases were used in this study: 2 cervical spine (C-spine) cases (case 2 and 7, Supplemental Digital Content 1, http://links.lww.com/CLINSPINE/A272) and 5 thoracic or lumbar spine (ThL-spine) cases (case 1, 3, 4, 5, and 6, Supplemental Digital Content 1, http://links.lww.com/CLINSPINE/A272). The participants were not familiar with the cases and were therefore blinded for the diagnosis and treatment given to the patient. The patients gave permission that their data could be used anonymously for research objectives. The diagnostic process and treatment considerations were investigated with the same seven questions for each case. Table 1 shows an overview of the questions of the case survey and the full case description of Case 1, Supplemental Digital Content 1, http://links.lww.com/CLINSPINE/A272. The full case descriptions of cases 1–7 are available online as Supplement 1, Supplemental Digital Content 1, http://links.lww.com/CLINSPINE/A272, including the key images of the cases at the time of presentation at the outpatient clinic.

**TABLE 1:** Course of the Survey; Each Case Had the Same Questions

*Case*	Anonymous description of the patient visiting the outpatient clinic Case 1 Female (age 57), 2 y after T11 A2 fracture treated with Jewitt brace. Current clinical presentation: disabling back pain irrespective of mobilization after pain free period, pain punctum maximum “bra-strap” and right side of trunk. Physical examination: mild kyphosis at “bra-strap,” no neurological deficit Additional: receives “Social security insurance” about disability benefits, DEXA scan showed no osteoporosis.
Q. 1	Which clinical parameters are most relevant in your decision to suspect SPTD? Please rank the parameters accordingly: 1 = most relevant; highest number = least relevant
Q. 2	What type of diagnostic assessment would you perform? And what is your requested information for each of the assessments? Local CR, full spine CR, flexion/extension CR, lateral bending CR, CT scan, MR scan, Nuclear imaging and diagnostic injections.
*Case*	Description of the assessments and their parameters, also the participants were able to view the radiologic assessments described in the description. Case 1 Trauma CT: T11 A2 fracture, posterior wall intact, no fractures in posterior structures, facets are aligned MRI +2 y: no edema in the bone or surrounding structures. Full spine AP and Lat: Cobb (T10-T12): 25 degree, ThK (T4-T12): 45 degree, ThL (T11-L1) 30 degree, LL (L1-L5): 74 degree, SS: 51 degree, PT: 25 degree, PI: 75 degree, SVA: 15 mm, Scoliosis lumbar 11 degree, some lumbar facet arthrosis.
Q. 3	Which diagnostic parameter is most relevant in your opinion to diagnose SPTD? Please rank the parameters accordingly: 1 = most relevant; highest number = least relevant Case 1 Trauma CT: level of fracture; MR scan; full spine: Cobb; full spine: ThK, ThL, LL; full spine: SS, PT, PI; full spine: SVA; full spine: Scoliosis; some lumbar facet arthrosis
Q. 4	What is missing (parameter or diagnostic assessment) for your decision to diagnose this case?
Q. 5	Is this a relevant Spinal Post-traumatic Deformity? Yes (If yes: asymptomatic OR symptomatic; compensated/balanced OR decompensated/imbalanced) No Not sure
Q. 6	What type of treatment would you consider? And what would that treatment be? Conservative treatment Surgical treatment Other
Q. 7	Do you have any additional remarks?

AP indicates anteroposterior; CR, conventional radiogram; Lat, lateral; LL, lumbar lordosis; PI, pelvic incidence; PT, pelvic tilt; Q, Question; SS, sacral slope; SVA, sagittal vertical alignment; ThK, thoracic kyphosis; ThL, thoracolumbar segment angle.

#### Question 1 Clinical parameters (ranking question)

After the case description, the participants were asked to rank the different aspects according to their relevance. The different categories were fracture type (history of the trauma and type of fracture), previous treatment, presence or absence of neurological deficit, complaints (pain, functional disability, etc.), physical examination performed, additional aspects (work status, PROMs), medical history (comorbidities), and sex/age. The lowest number corresponded to being most relevant, the highest number to the least relevant.

#### Question 2 Diagnostic assessment

The presented diagnostic assessments (local CR, full spine CR, flexion/extension CR, lateral bending CR, computed tomography (CT) scan, magnetic resonance (MR) scan, nuclear imaging, and diagnostic injections) were based on the information gathered from the systematic review and the previously conducted survey.^6,7^

#### Question 3 Diagnostic assessment (ranking question)

After presentation of the different diagnostic assessments including images and measurements, the participants were asked to rank the different groups of parameters from most to least relevant. The groups were trauma (fracture type and configuration), local deformity (Cobb and wedge angles at presentation), global alignment (thoracic kyphosis, thoracolumbar Cobb angle, and lumbar lordosis), sagittal balance (SVA and cervical SVA), and pelvic parameters (pelvic tilt, sacral slope, and pelvic incidence) and additional (facet arthrosis, osteophytes, and absence of myelopathy).

#### Question 4 Missing additional information

From the previously held studies and the face-to-face meetings with the KF Trauma, it was clear that there is a wide variation on the perceptions of SPTD. We wanted to give the opportunity to add anything that might be important to form their diagnosis.

#### Question 5 Is this SPTD?

The participants were asked if the patient had SPTD, and if present, if it was asymptomatic or symptomatic SPTD, and if it was compensated or decompensated SPTD. For example, a compensated patient may have a deformity and complaints, but was able to maintain sagittal alignment.

#### Question 6 Treatment

To evaluate the different treatment options used by the participants, they could choose their preferred treatment for that case and specify what that treatment entailed.

#### Question 7 Additional remarks

Participants could add any additional remark to specifics of the case or in general to make sure no important details were missing.

#### Data Collection and Analysis

The survey was distributed with REDcap (REDCap Software—Version 6.5.2 2020 Vanderbilt University) and the images were anonymized and distributed in Surfdrive (Coöperatie SURF U.A., the Netherlands). Distribution was between May 1 and July 31. The researcher doing the analysis was blinded by the identity of the participants of the survey. R statistical software (R version 3.3.2; R Foundation for Statistical Computing, Vienna, Austria) was used for the analysis of descriptive statistics. The data from questions 1 and 3 were normalized to a 0–100 scale to compare the cases. Consensus was reached when ≥80% of experts agreed.^8–11^

## RESULTS

### Demographics

In total 31 spine surgeons received the case survey, which was completed by 17 (55%). The KF Trauma was represented by 13 participants who completed the full survey and the KF Deformity by 2 participants. In addition, 3 surgeons specialized in spine deformity completed the full survey and 1 spine surgeon completed only the first case. All participants had >5 years of experience as spine surgeons.

### Clinical Parameters

**FIGURE 1:**
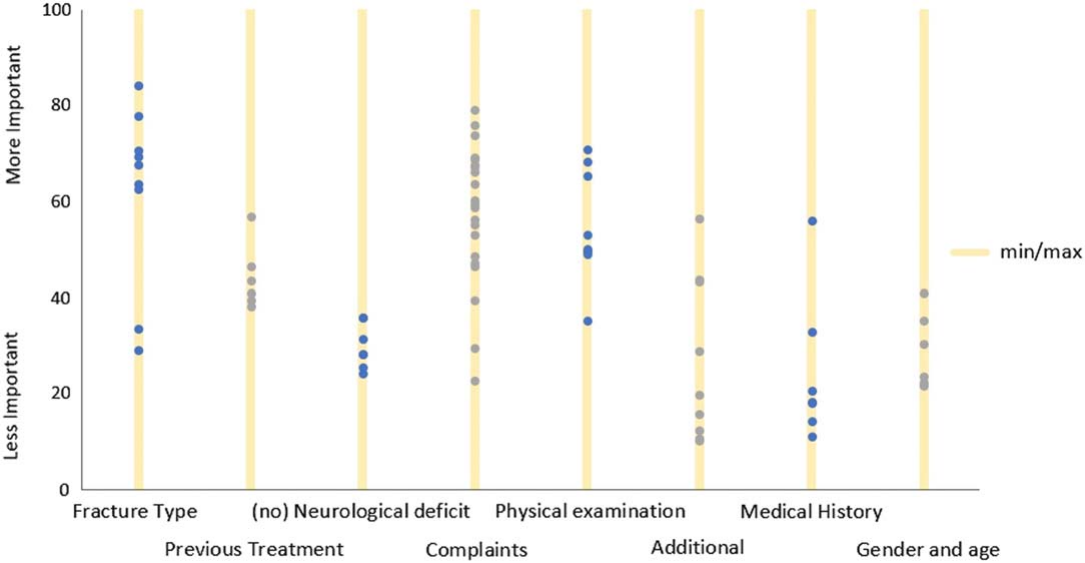
Distribution of the different clinical aspects of the 7 cases. This figure shows the distribution of the different clinical aspects of the 7 cases. All aspects were rated as most and least important at least by 1 participant. No consensus was reached for the items overall or for the individual cases.

The results are depicted in Figure 1. The items’ fracture type and complaints tended to be rated by the participants as more important. The items’ additional medical history, sex/age, and neurology tended to be rated as less important. All aspects were rated as most and least important at least by 1 participant. No consensus was reached for the items overall or for the individual cases.

### Diagnostic Assessment

See Table 2 for the results and reasons for the requested assessments. For the C-spine cases (case 2 and 7, Supplemental Digital Content 1, http://links.lww.com/CLINSPINE/A272) the majority of the participants requested a cervical CR (76%–83%), a flexion/extension CR (61%–71%), a CT scan (76%–89%) and a MR scan (89%–94%). A full spine CR (89%–100%), a CT scan (72%–94%), and a MR scan (65%–94%) were requested most in the ThL-spine cases (cases 1 and 3–6, Supplemental Digital Content 1, http://links.lww.com/CLINSPINE/A272). The imaging was, however, partly requested for surgical planning purposes and not solely for diagnostic purposes.

**TABLE 2:** Diagnostic Assessment Who Were Requested by the Participants

Diagnostic Assessment	Region	Request percentage	Reasons for request
Local CR	C	76–83	Local deformity/collapse, cervical alignment, K-line, disc damage
	ThL	33–47	Local deformity/collapse, other pathology, instability/progression, instrumentation placement
Full spine CR	C	24–44	Sagittal and coronal balance, thoracic deformity
	ThL	89–100	Sagittal and frontal balance, pelvic parameters, (progression of) local deformity, regional/global alignment, surgical planning, scoliosis, instrumentation placement
Flexion/extension CR	C	61–71	Stability, mobility fracture, reduction possible, pseudo arthrosis
	ThL	24–53	Stability, mobility fracture, compensation, pseudo arthrosis
Lateral bending CR	C	0	—
	ThL	0–6	Instability
CT scan	C	76–89	Injury details, fusion/nonunion/pseudo arthrosis, surgical planning, bone quality, facet alignment
	ThL	72–94	Bone quality, nonunion/pseudo arthrosis/healing, facet joints, anatomy, surgical planning, anatomy, screw integrity, and positioning
MR scan	C	89–94	Compromised neurological structures, PLC-injury, status of discs, stenosis, nonunion/healing, other pathology, stenosis
	ThL	65–94	Compromised neurological structures, PLC-injury, status of discs, stenosis, nonunion/healing, surgical planning
Nuclear imaging	C	0	—
	ThL	0–6	Bone health
Diagnostic injections	C	0	—
	ThL	0–44	Discern between source of pain: discography to detect disc problems, degenerative pain source
Other	C	0	
	ThL	0–18	BMD/DEXA scan

C indicates cervical; CR, conventional radiograph; PLC, posterior ligamentous complex of the spine; ThL, thoracolumbar.

### Diagnostic Assessment of Imaging Parameters

**FIGURE 2:**
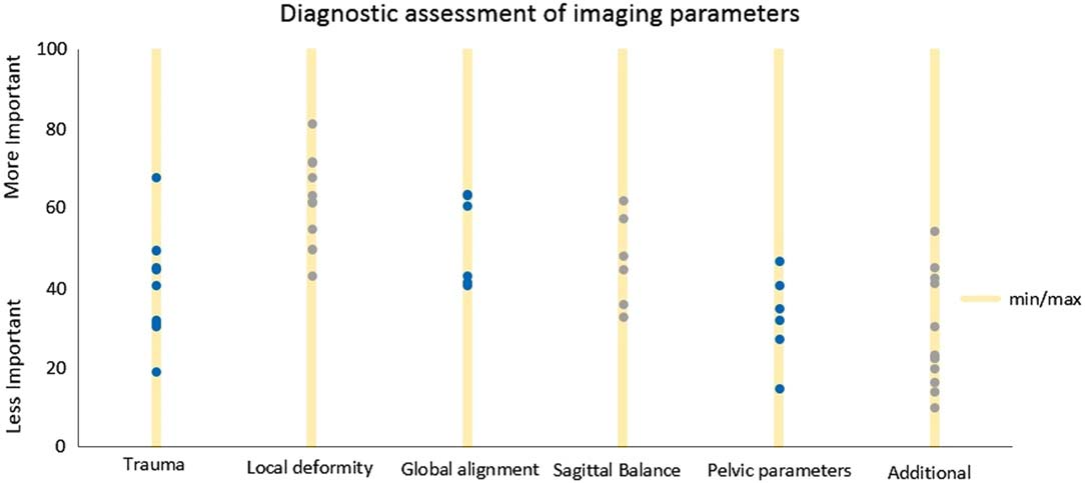
Distribution of the different aspects of the imaging of the 7 cases. This figure shows the distribution of the ratings of the different aspects of the imaging assessments. The participants were asked to rank from most to least important. All groups were rated at least once as most important and least important by 1 participant. No consensus was reached overall or in the individual cases.

In Figure 2, the results are depicted. The local deformity tended to be ranked as more important, the pelvic parameters and the additional parameters to be less important. All groups were rated at least once as most important and least important by 1 participant. No consensus was reached overall or in the individual cases.

### Additional Information Wanted for Diagnosis

Not all the cases had all the requested assessments available. Four diagnostic assessments were deemed missing in C-spine cases: cervical CR, flexion/extension CR, full spine CR, and MRI at the time of trauma. In the ThL-spine cases additional information on pain behavior pattern/psychiatric evaluation, diagnostic injections, supine and standing CRs, description of gait pattern, bone quality measurement, lordosis distribution index, and Oswestry disability index was wanted by the experts.

### Relevant SPTD

**FIGURE 3:**
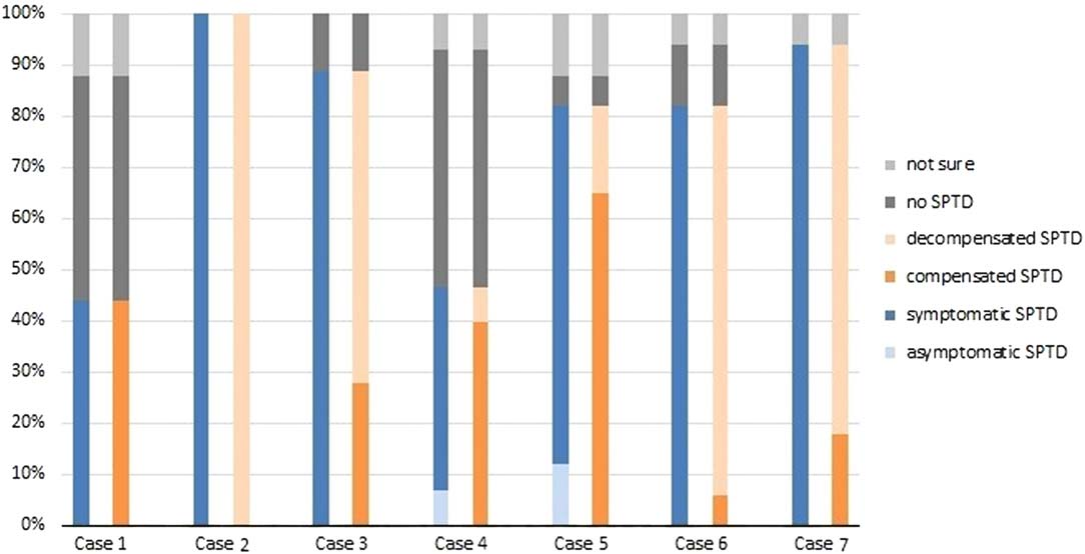
Presence and type of SPTD per case. This figure shows whether the participants diagnosed the patients with SPTD per case. Consensus was present on the case being compensated or decompensated and being symptomatic or asymptomatic, when there was agreement on the presence of SPTD. SPTD indicate Spinal Post-traumatic Deformity.

Five out of the seven cases (cases 2, 3, 5, 6, and 7, Supplemental Digital Content 1, http://links.lww.com/CLINSPINE/A272) were classified as SPTD (82%–100%) and in 2 other cases (cases 1 and 4, Supplemental Digital Content 1, http://links.lww.com/CLINSPINE/A272) no consensus was reached among the participants (35%–44%). In the 5 cases in which consensus was reached, there was also consensus on the case being compensated or decompensated and being symptomatic or asymptomatic. Figure 3 shows the opinion of the participants per case on the presence and type of SPTD.

### Treatment

**FIGURE 4:**
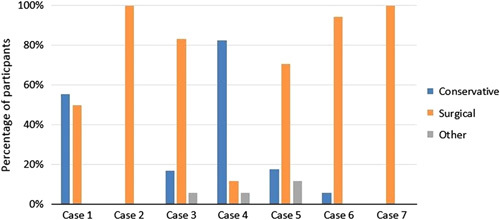
Treatment choices per case. This figure shows the distribution of the treatment choices of the participants per case.

The participants unanimously agreed that they would treat both the cervical cases (cases 2 and 7, Supplemental Digital Content 1, http://links.lww.com/CLINSPINE/A272) surgically. Only the thoracolumbar cases with a higher agreement toward SPTD (cases 3 and 6, Supplemental Digital Content 1, http://links.lww.com/CLINSPINE/A272) would be treated surgically by most participants. There was consensus to treat case 4, Supplemental Digital Content 1, http://links.lww.com/CLINSPINE/A272 conservatively, without agreement on SPTD. Figure 4 shows the distribution of treatments per case.

### Other Comments

The additional comments were mainly on the treatment contemplations of the cases. Those contemplations depended, for example, on the effect of diagnostic injections before surgery or whether a conservative therapy would be initiated first because of the short time between trauma and the presentation of the patient. Also, comments on missed fractures were mentioned such as: “this is not SPTD, but B-type injury possibly missed.”

## DISCUSSION

The current study is unique and innovative in the field of spine trauma and has been set up as a preparatory study for a future Delphi study. This study showed the clinical path of patients with sequels after a spine trauma and the different opinions of spine experts in their diagnosis and treatment. There was a strong consensus in 5 out of 7 cases on the diagnosis of clinically relevant SPTD. There was also strong consensus on the use of specific imaging assessments. For C-spine cases: cervical CR, CT scan, and MR scan. For ThL-spine cases: full spine CR, CT scan, and MR scan.

The diagnostic assessments were requested by the participants for many different reasons (Table 2). When constructing this survey, we decided to split the diagnostic assessment from the treatment considerations. The responses from our participants showed that some imaging modalities serve both diagnostic and treatment planning. In current medical practice, treatment is considered from the moment a patient enters the room of the surgeon. We should strive for the minimum of imaging modalities necessary, but refrain from repeating similar modalities without receiving additional information for the diagnosis or treatment of the patient.

Earlier studies suggested that asymptomatic SPTD can be present in patients after a spine trauma.^6,12^ Patients with an asymptomatic SPTD might not seek the advice of the spine surgeon because there are no complaints. However, with increasing age the compensation mechanisms in place might decline and an asymptomatic patient can present with symptoms over time.^13^ There was consensus that patients with asymptomatic SPTD should not receive surgical treatment. This was in line with the opinion of Boehm et al.^13^ They also stated that patients without complaints should be monitored closely and informed of the possibility of a decline in mobility and the development of arthrosis.^13^

Our study shows that in most cases unanimous agreement was achieved that the patients exhibited a clinically relevant SPTD. However, the participants did not agree on 2 ThL-spine cases (cases 1 and 4, Supplemental Digital Content 1, http://links.lww.com/CLINSPINE/A272). In the results of those 2 cases, we found differing opinions and wide ranges concerning questions 1 and 4. Subsequently, there was relatively less deformity visible on the diagnostic imaging compared with the cases with consensus on the diagnosis of SPTD. This highlights the problem that disagreement exists on the edges of the spectrum of SPTD, and surgeon variability, preference, and available resources are part of this disagreement.

Some specific comments raised questions about a missed fracture without proper treatment. This is an interesting standpoint about the causes of this type of spinal deformity. Is there a categorical difference between missed fractures and fractures that were diagnosed and treated, if they end up in similar symptomatic deformities?

Our study was limited by the number of listed factors of the visit at the outpatient clinic. At the outpatient clinic, the patient’s history and physical examination were noted by a physician. But not all patients will have the same amount of information noted from the visit. For example, the participants could rank 9 different factors for case 4, Supplemental Digital Content 1, http://links.lww.com/CLINSPINE/A272, and up to 13 factors for case 2, Supplemental Digital Content 1, http://links.lww.com/CLINSPINE/A272. We decided not to create equal amounts of factors because this would mean withholding possible important pieces of information. A complete standardized approach could have led to greater consensus, but if we do not know which parameters are the most essential it is difficult to set up such an approach. To allow comparison the data from the different factors was normalized during analysis to a 0–100 score.

Additional to the case variability, several surgeon-specific factors could contribute to the overall disagreement in this study. For example, some variability can be attributed to the resources available to the surgeon, the experience, and the preference of the surgeon. This could be prevented by choosing a specific population of surgeons; however, in our aim to create an international consensus definition, we thought it important to include an international group of surgeons.

The strength of our study lies in the fact that this survey was based on real cases as they presented themselves at the outpatient clinic. This strategy enabled us to capture the diagnostic process of the spine surgeon and see how decisions were being made during that process. We highlighted that patients with possible SPTD did not receive the proper treatment at first or that injury was missed. This confirms that SPTD is a preventable complication of spine trauma. To prevent this complication, it is essential to know the risk factors but to study these, a proper definition of clinically relevant SPTD is necessary.

The findings from this study add to the growing understanding of STPD. Consensus on which imaging assessments to use and consensus on certain cases helps us in the search for a consensus definition of clinically relevant SPTD. Our next steps will be to perform a Delphi study among the global spine community to create this consensus definition of clinically relevant SPTD.

## Supplementary Material

## References

[1] Sadatsune DA Costa PP Caffaro MFS . Thoracolumbar burst fracture: correlation between kyphosis and function after surgical treatment. Rev Bras Ortop. 2015;47:474–478. 27047853 10.1016/S2255-4971(15)30131-2 PMC4799454

[2] Verlaan JJ Diekerhof CH Buskens E . Surgical treatment of traumatic fractures of the thoracic and lumbar spine: a systematic review of the literature on techniques, complications, and outcome. Spine (Phila Pa 1976). 2004;29:803–814. 15087804 10.1097/01.brs.0000116990.31984.a9

[3] Vaccaro AR Silber JS . Post-traumatic spinal deformity. Spine (Phila Pa 1976). 2001;26(suppl 24):S111–S118. 11805617 10.1097/00007632-200112151-00019

[4] Polly DWJ Klemme WR Shawen S . Management options for the treatment of posttraumatic thoracolumbar kyphosis. Semin Spine Surg. 2000:110–116.

[5] Buchowski JM Kuhns CA Bridwell KH . Surgical management of posttraumatic thoracolumbar kyphosis. Spine J. 2008;8:666–677. 17662662 10.1016/j.spinee.2007.03.006

[6] De Gendt EEA Vercoulen TFG Guo W . The current status of Spinal Posttraumatic Deformity: a systematic review. Global Spine J. 2021;11:1266–1280. 33280414 10.1177/2192568220969153 PMC8453678

[7] De Gendt EEA Schroeder GD Joaquim A . Spinal posttraumatic deformity: an international expert survey amongst AO Spine Knowledge Forum members. Clin Spine Surg. 2020;36:E94–E100. 10.1097/BSD.0000000000001376 35994038

[8] Keeney S Hasson F McKenna H . Consulting the oracle: ten lessons from using the Delphi technique in nursing research. J Adv Nurs. 2006;53:205–212. 16422719 10.1111/j.1365-2648.2006.03716.x

[9] Hasson F Keeney S McKenna H . Research guidelines for the Delphi survey technique. J Adv Nurs. 2000;32:1008–1015. 11095242

[10] Hung H-L Altschuld JW Lee Y-F . Methodological and conceptual issues confronting a cross-country Delphi study of educational program evaluation. Eval Program Plann. 2008;31:191–198. 18403018 10.1016/j.evalprogplan.2008.02.005

[11] de Kleuver M Faraj SSA Holewijn RM . Defining a core outcome set for adolescent and young adult patients with a spinal deformity: a collaborative effort for the Nordic Spine Surgery Registries. Acta Orthop. 2017;88:612–618. 28914116 10.1080/17453674.2017.1371371 PMC5694805

[12] Schoenfeld AJ Wood KB Fisher CF . Posttraumatic kyphosis: current state of diagnosis and treatment: results of a multinational survey of spine trauma surgeons. J Spinal Disord Tech. 2010;23:e1–e8. 20124914 10.1097/BSD.0b013e3181c03517

[13] Boehm H Shousha M Korrekturosteotomie RB . Correction Osteotomies for posttraumatic misalignments [für posttraumatische Fehlstellungen]. Trauma und Berufskrankheit. 2017;19:86–96.

