# Supplementary material for: The Diagnostic Process of Spinal Post-traumatic Deformity: An Expert Survey of 7 Cases, Consensus on Clinical Relevance Does Exist
Source: Clin Spine Surg. 2023 Jun 26;36(8):E383–9. doi: 10.1097/BSD.0000000000001478 (PMC10521791; doi:10.1097/BSD.0000000000001478)

**Supplement 1. Case descriptions**

***Case 1***

Visit outpatient clinic

Female, age 57

2 years after T11 A2 fracture. Received conservative treatment with Jewitt brace.

Current clinical presentation: Disabling back pain irrespective of mobilization after pain free period, pain punctum maximum ‘bra-strap’ and right side of trunk.

Physical examination: mild kyphosis at ‘bra-strap’, no neurological deficit

Additional: receives 'Social security insurance' about disability benefits, DEXA-scan showed no osteoporosis

Radiological assessments and parameters

Trauma CT: T11 A2 fracture, posterior wall intact, no fractures in posterior structures, facets are aligned

MRI +2years: no edema in the bone or surrounding structures.

Full spine AP and Lat: Cobb (T10-T12): 25˚, ThK (T4-T12): 45˚, ThL(T11-L1) 30˚, LL (L1-L5): 74˚, SS: 51˚, PT: 25˚, PI: 75˚, SVA: 15mm, Scoliosis lumbar 11˚, some lumbar facetarthrosis.

| 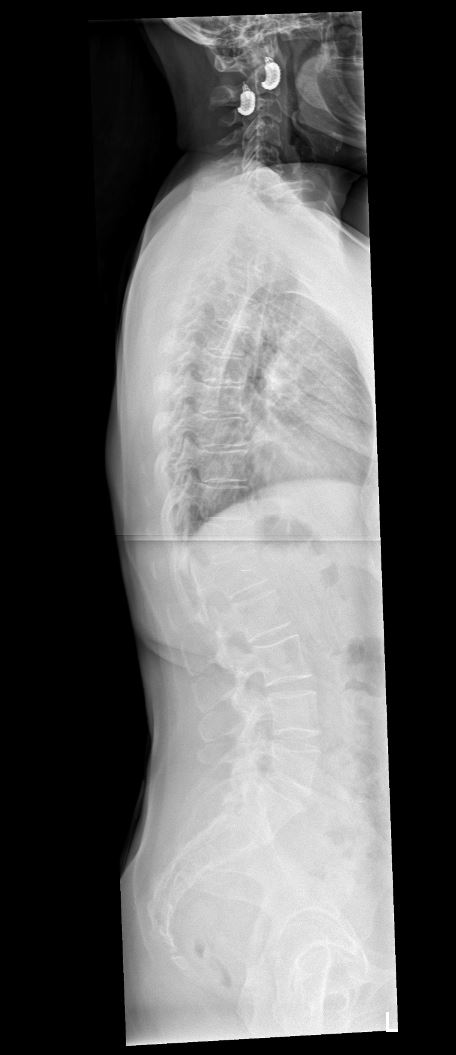 | 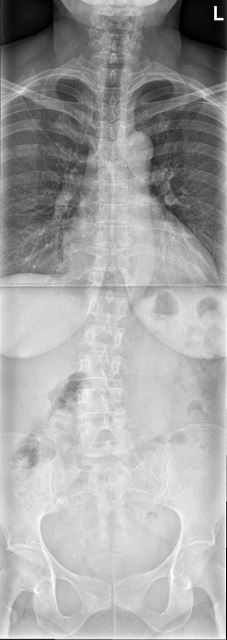 |
| --- | --- |
|  |  |

***
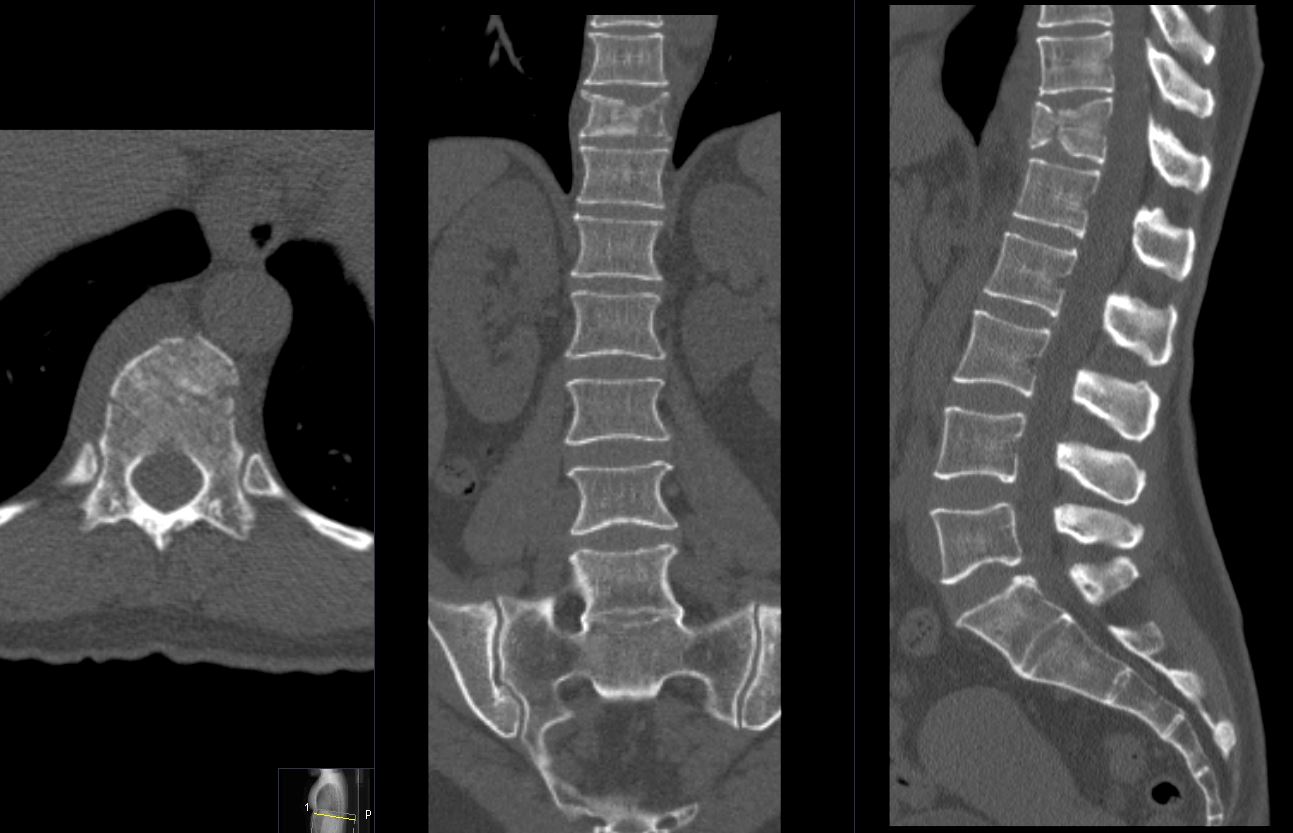
***

***
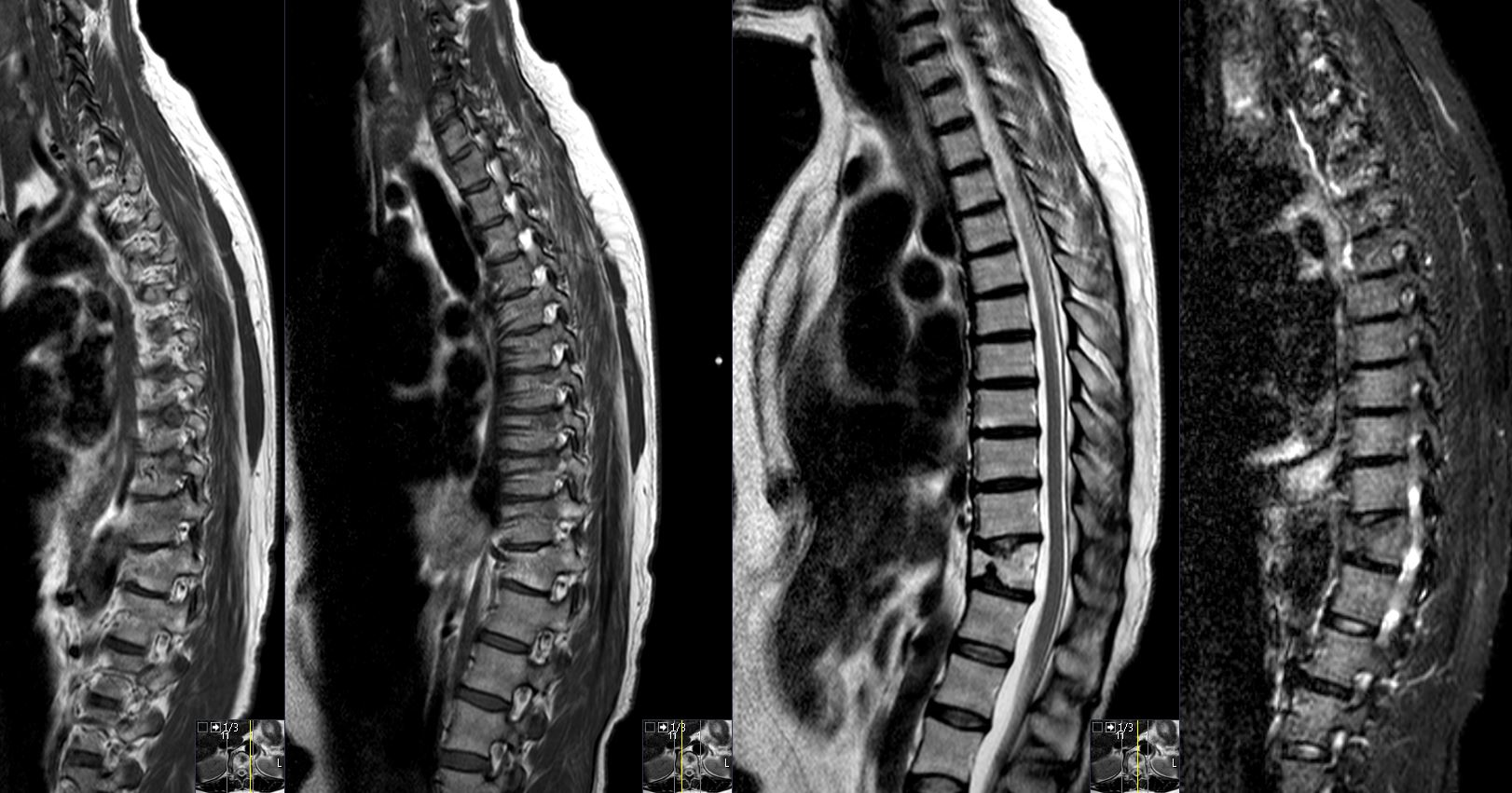
***

***Case 2***

Visit outpatient clinic

Male, age 68

Presentation 6 months after fall from small step on back/neck, with 2 weeks temporary paresthesia in right arm, no injury on trauma CT. Received no treatment except physiotherapy for contusion neck.

Current clinical presentation: Pain right side of neck and shoulder (NRS pain 7/10), exacerbated by coughing/sneezing, unable to play tennis. Head falls forward.

Physical examination: pain at level C6, no neurological deficit

Additional: No medical history. Work: retired, Neck Disability Index: 13 (minimal disability), AOSpine PROST: 79/100 (total)

Radiological assessments and parameters

CT trauma: no evident fracture, enlarged facet joint C5-6 Right>Left

Cervical radiograph: Cobb (C4-C6): 32˚(kyphosis), wedge (C6): 2˚, C2-7 L: 21 ˚(kyphosis), C2-7 SVA: 28mm, T1 slope: 21˚

MRI +6months: injury disc/PLC at level C5-6 with major dislocation/kyphosis, no evident myelopathy

CT +6months: Major anterior dislocation C5-6, with bilateral dislocation of the facet joints, anterior osteophytes present at C5-6.


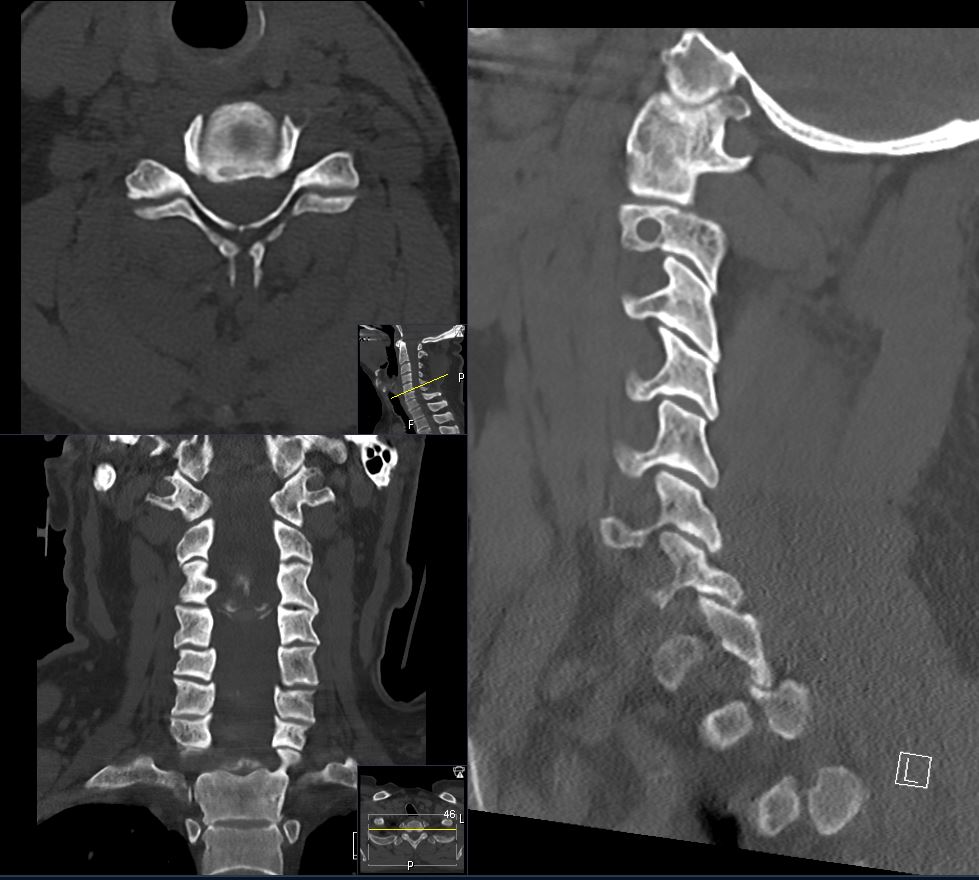


| 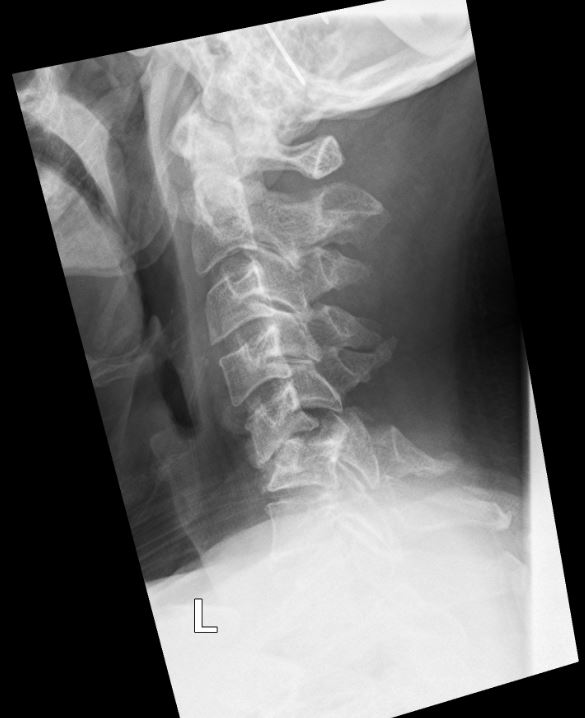 |  |
| --- | --- |


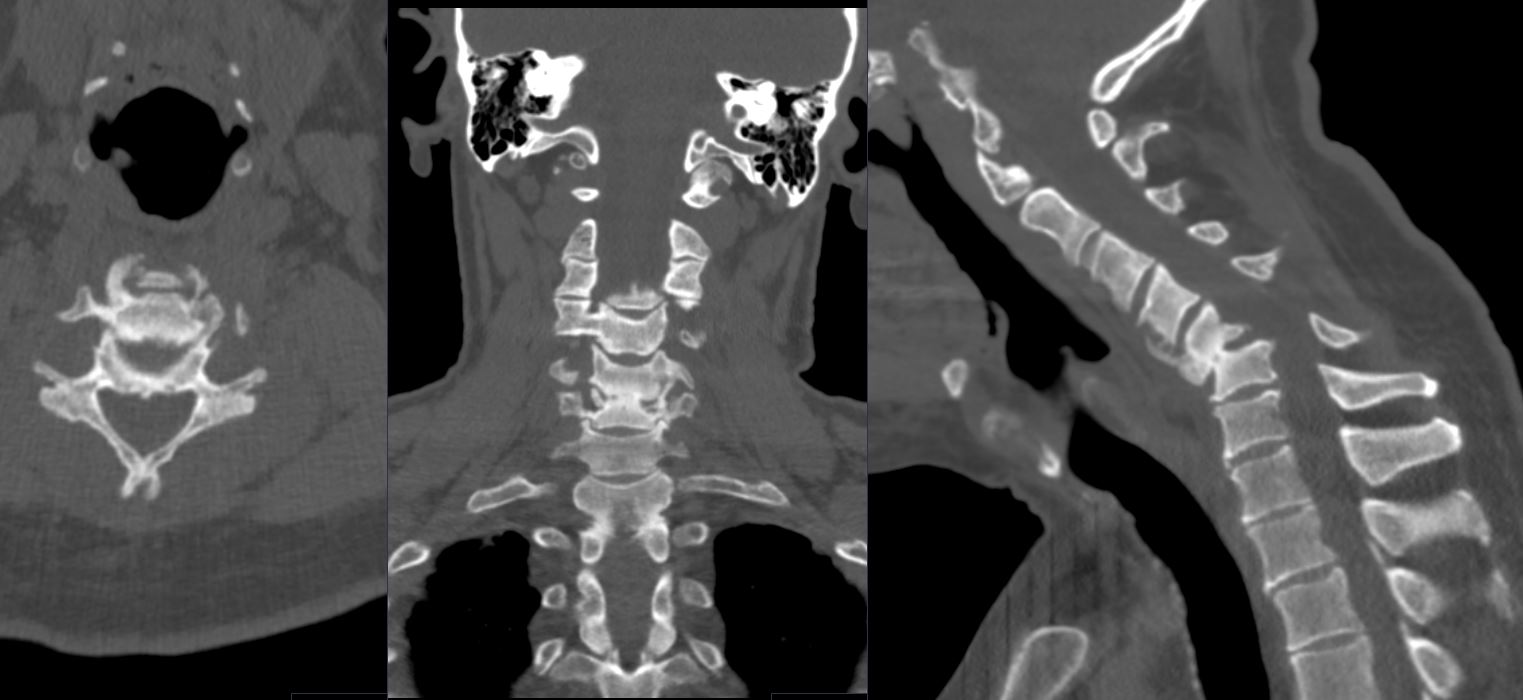


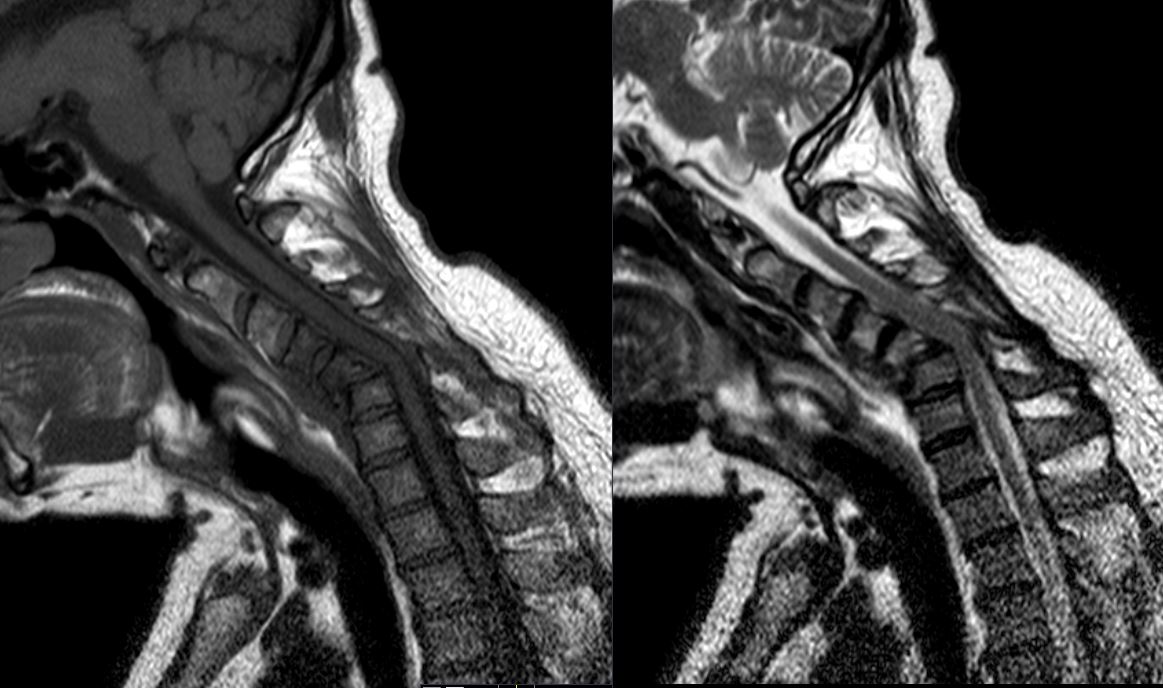


***Case 3***

Visit outpatient clinic

Male, age 64

Presentation 4 moths after fall from height(2.5m), L3 A4 fracture. Received conservative treatment with brace for 6 weeks. Also suffered a distal radius fracture.

Current clinical presentation: After stop brace increasing pain lower back, problems with lifting upper right leg, no incontinence.

Physical examination: Absent lumbar lordosis, hyperkyphotic spine, MRC 5- left quadriceps, remaining MRC 5, no other neurological deficit

Additional: No medical history. Work: self-employed builder, unable to work

Radiological assessments and parameters

CT trauma: L3 A4 fracture, minimal protrusion in the canal

MRI T2 sag +4 months: no neural compression, relative canal stenosis.

Full spine AP and Lat: Cobb (L2-L4): 9˚, wedge (L3): 35˚, ThK (T4-T12): 36˚, ThL(T11-L1) 4˚, LL (L1-L5): 24˚, SS: 22˚, PT: 30˚, PI: 52˚, SVA: 67mm, scoliosis Cobb 15˚


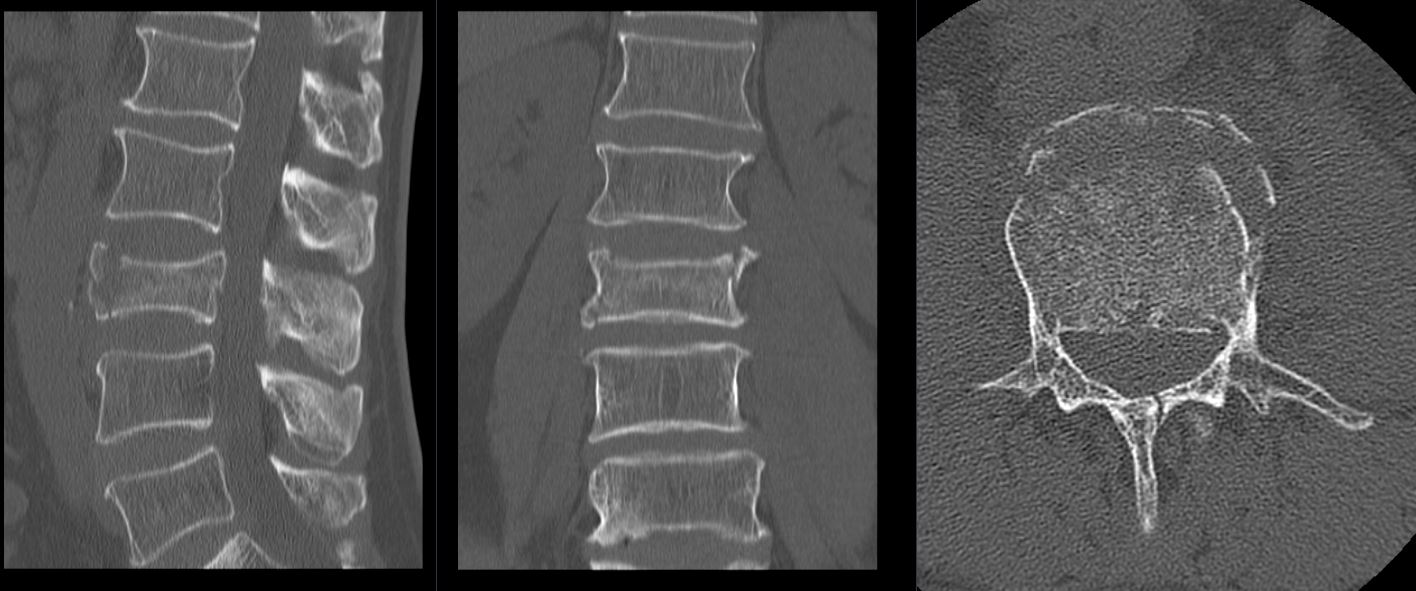

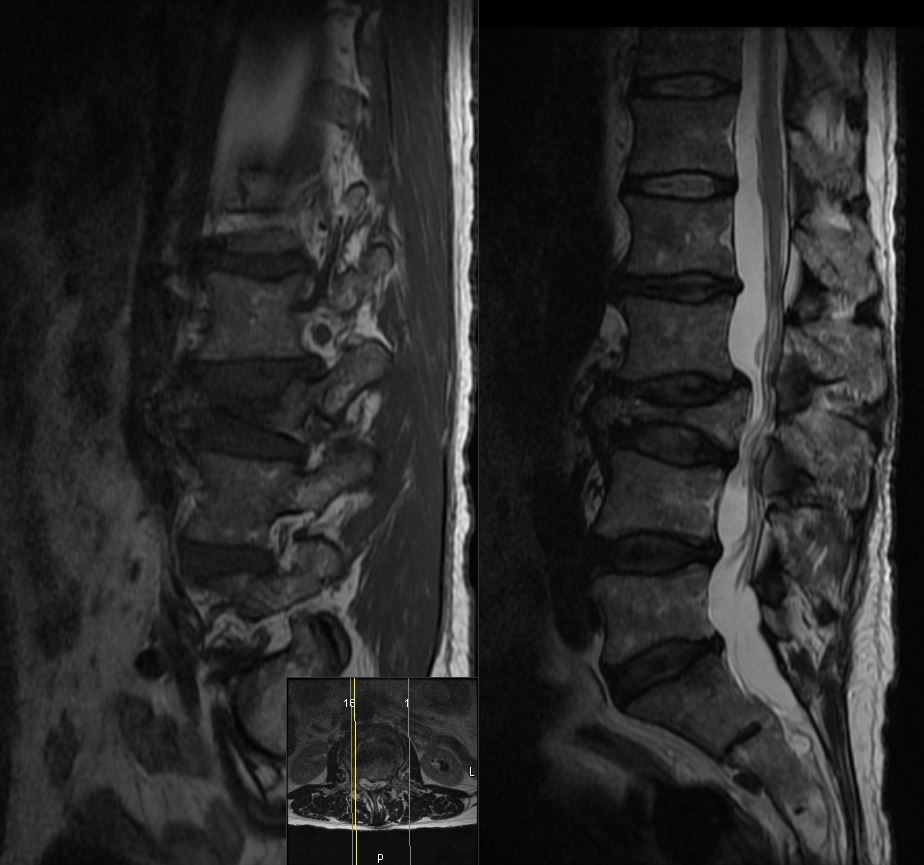


| 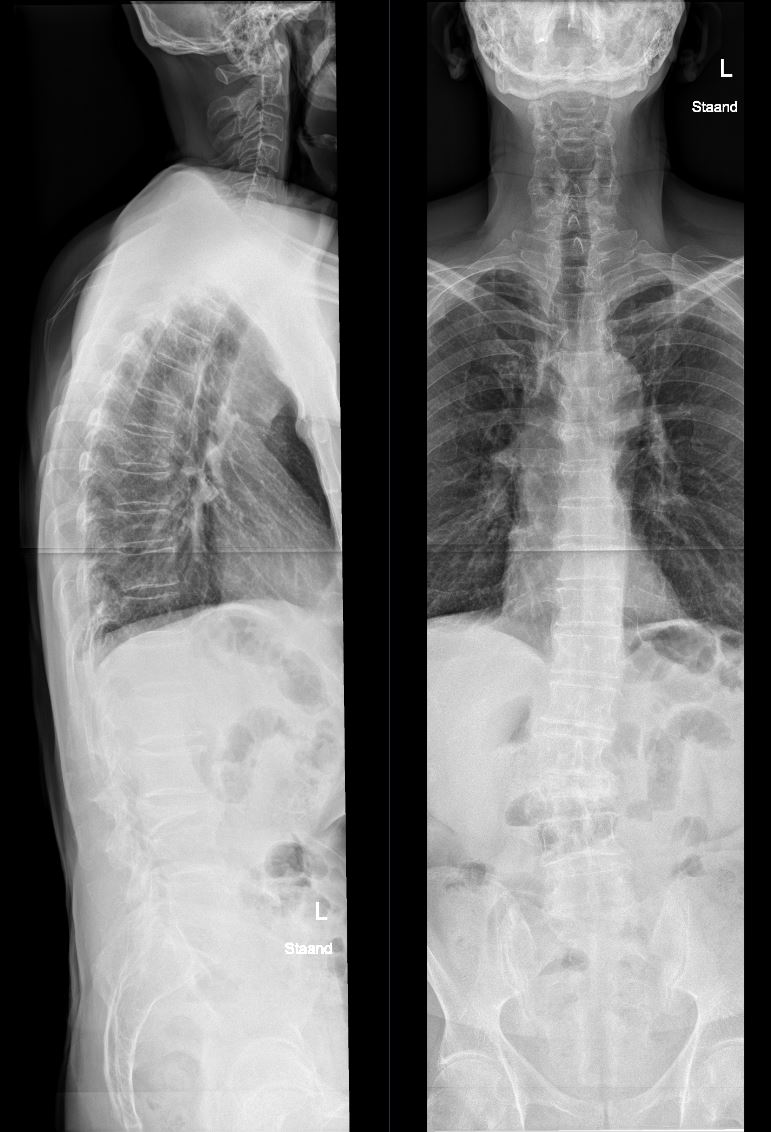 |
| --- |

***Case 4***

Visit outpatient clinic

Female, age 59

Presentation 1 year after pedestrian vs car (30 km/h), T10 A2 fracture. Received conservative treatment with mobilization and painkillers. Other injuries: Facial fractures, right subcapital humerus fracture, right sacral and acetabular fracture (all conservatively treated).

Current clinical presentation: Diffuse back and neck pain mostly after exertion

Physical examination: Palpation painful mostly paravertebral, no neurological deficit

Additional: Hypertension, Diabetes Mellitus 2. Work: Social worker psychiatry/geriatrics

Radiological assessments and parameters

CT trauma: T10 A2 fracture

MRI +1month: no ligamentous injury

Full spine AP + Lat +4months: Cobb (T9-T11): 20˚, wedge (T10): 19˚, ThK (T4-T12): 46˚, ThL(T11-L1) -1˚, LL (L1-L5): 60˚, SS: 39˚, PT: 15˚, PI: 53˚, SVA: 39mm

Thoracic and Lumbar spine sag +1 year: Cobb (T9-T11): 20˚, wedge (T10): 20˚


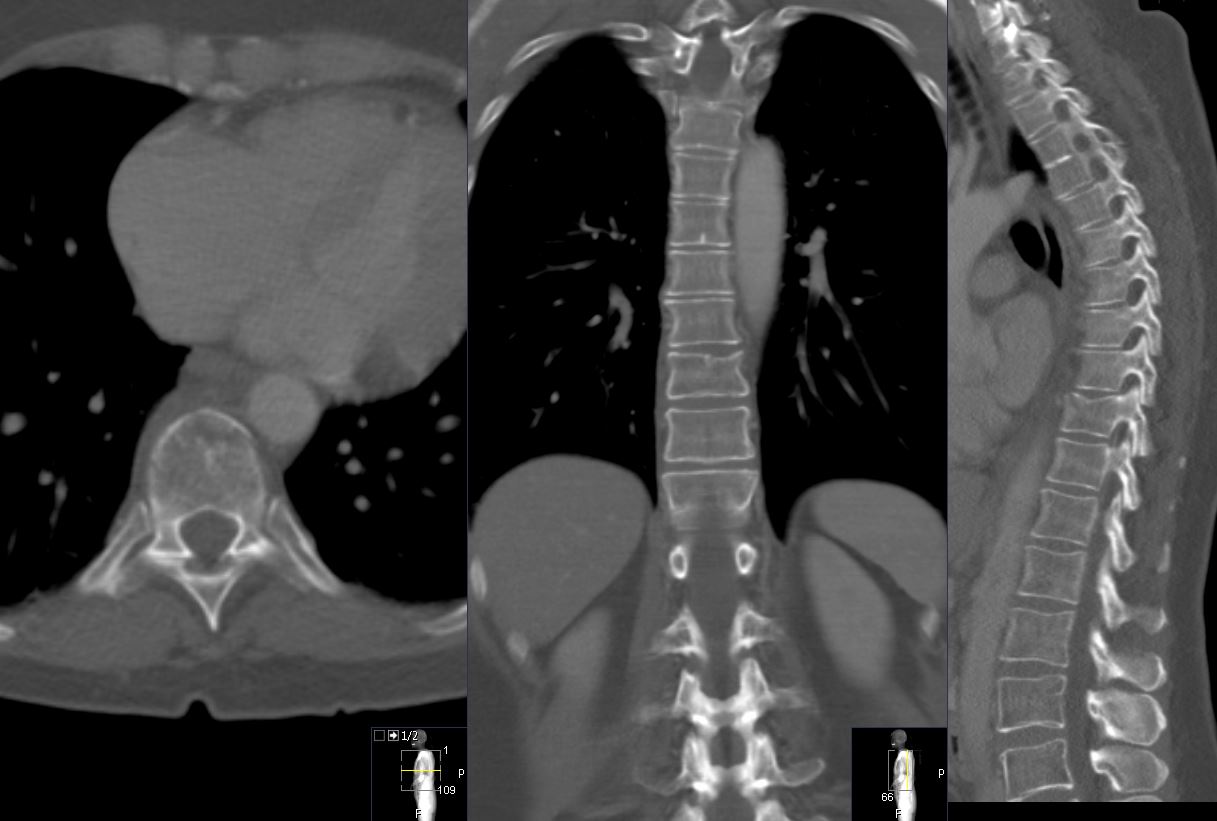


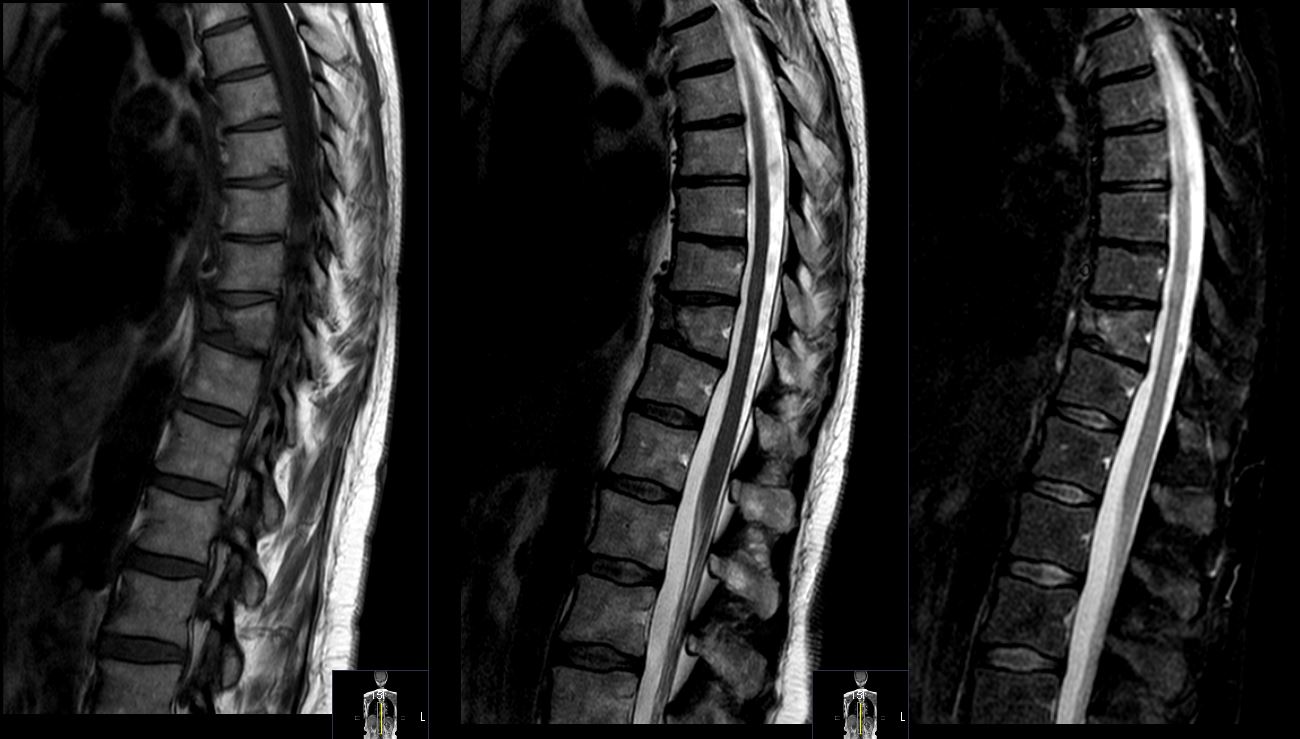


| 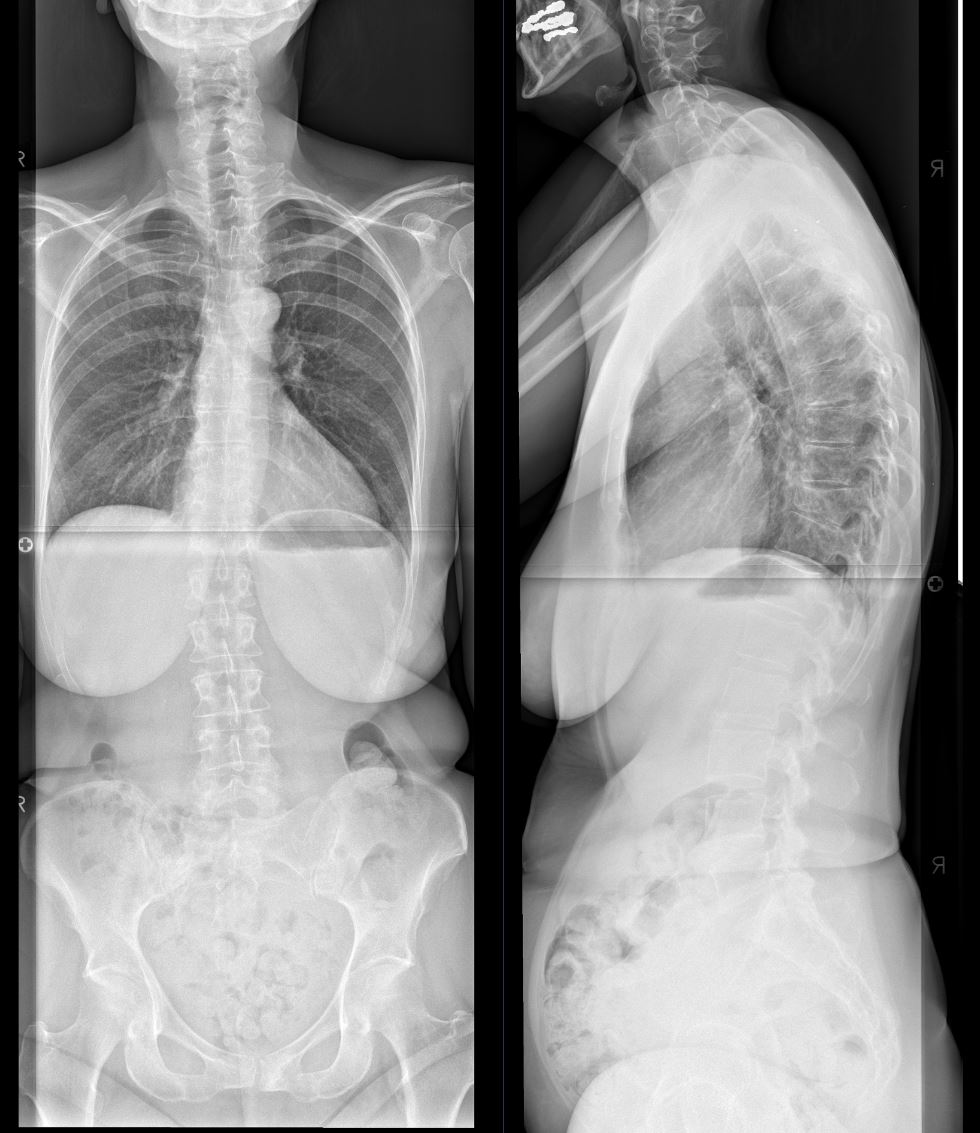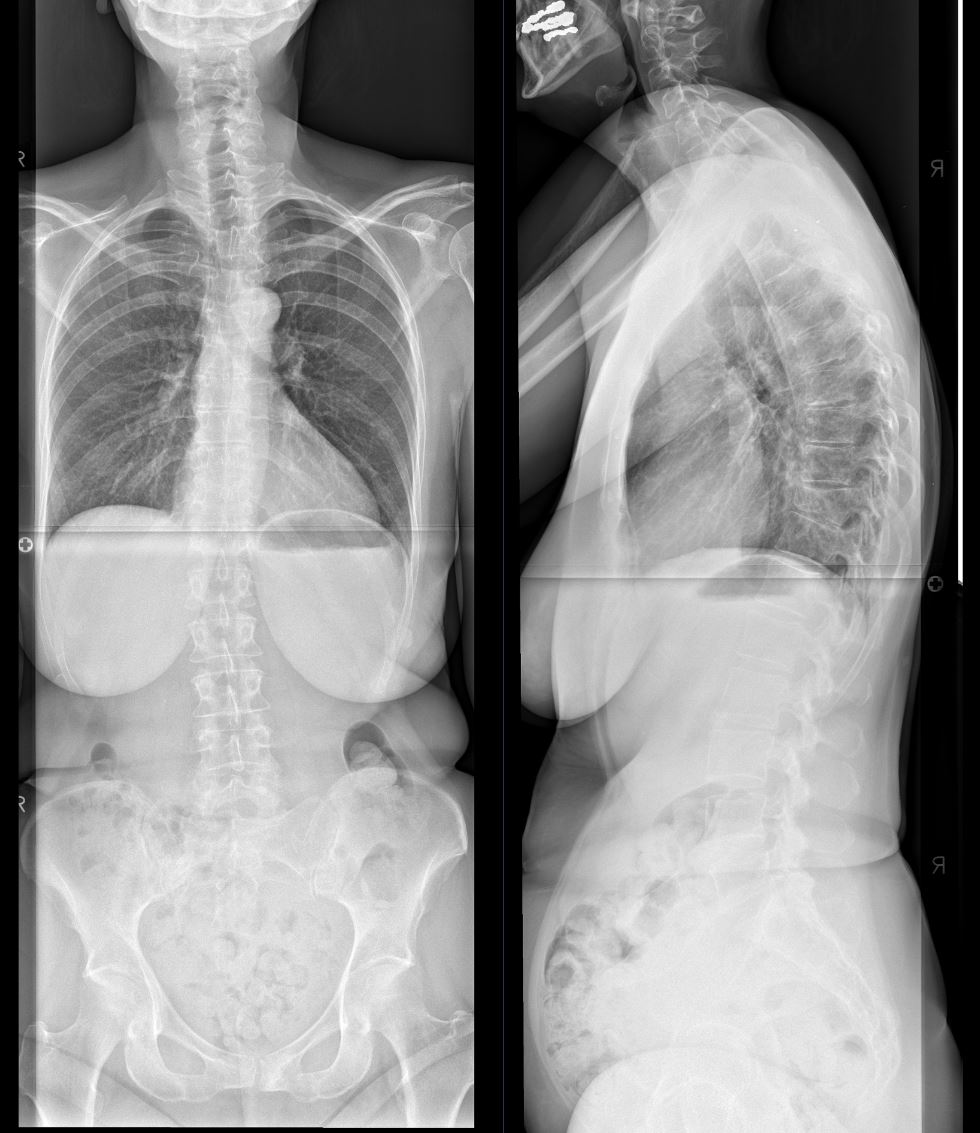 |
| --- |

***Case 5***

Visit outpatient clinic

Male, age 67

Presentation 4 months after bike accident, radiograph T4/5 compression/burst fractures (no CT-scan of thoracic spine), received conservative treatment with painkillers, mobilization and physiotherapy. Other injury: sternal fracture

Current clinical presentation: Persistent chest pain, pain between shoulder blades after 30-45min walking/biking.

Physical examination: No pain on palpation back, evident sternal offset, no neurological deficit

Additional: hypercholesterolemia. Work: retired (carpenter, still practices)

Radiological assessments and parameters

Tspine lat: T4 A3, B component cannot be identified

Full spine AP+lat +4months: Cobb (T3-T5): 46˚, wedge (T4): 23˚, wedge (T5): 22˚, ThK (T4-T12): 57˚, ThL(T11-L1) 12˚, LL (L1-L5): 53˚, SS: 29˚, PT: 16˚, PI: 46˚, SVA: -8mm

CT +4 months: T4 A4B2, T5 A3, T11 A1, sternal fracture; Vacuum cleft T4 and T5.

MRI +4 months: no myelopathie, some bone marrow oedema on STIR

| 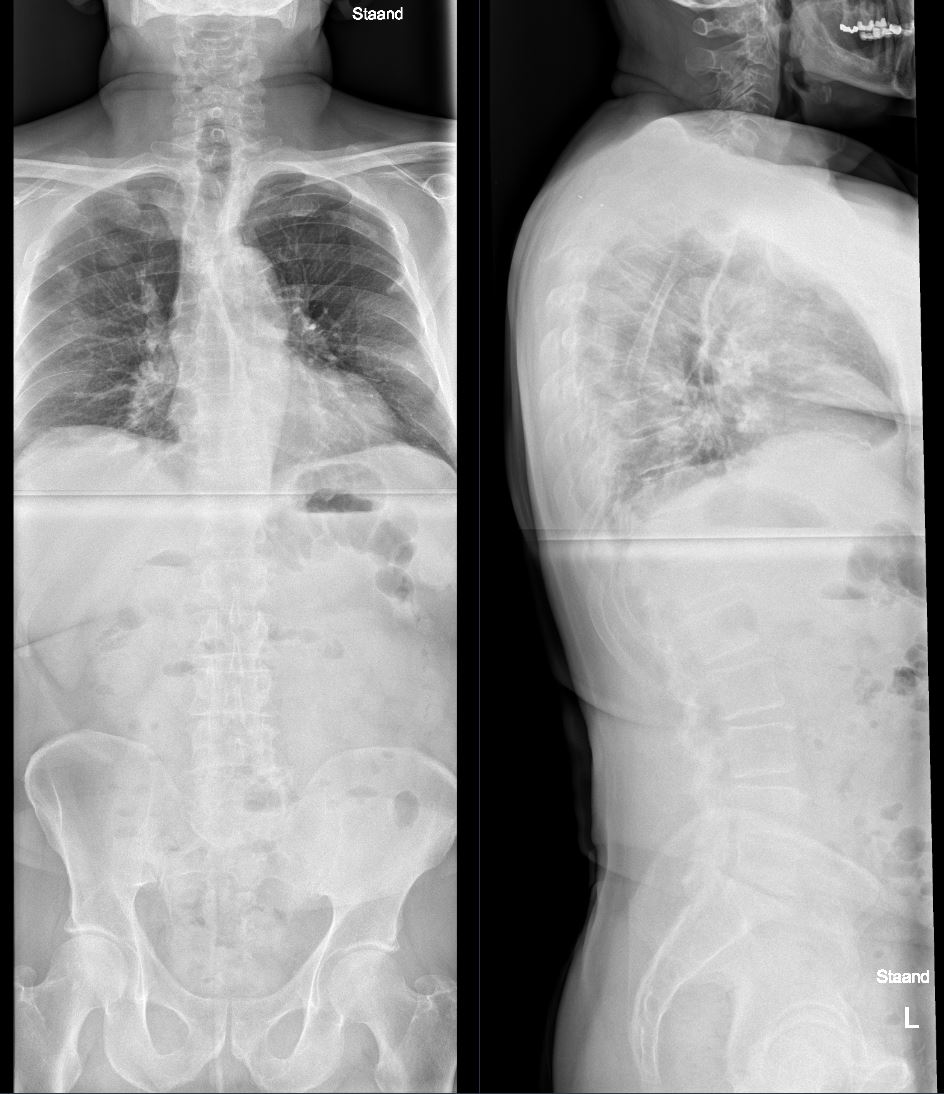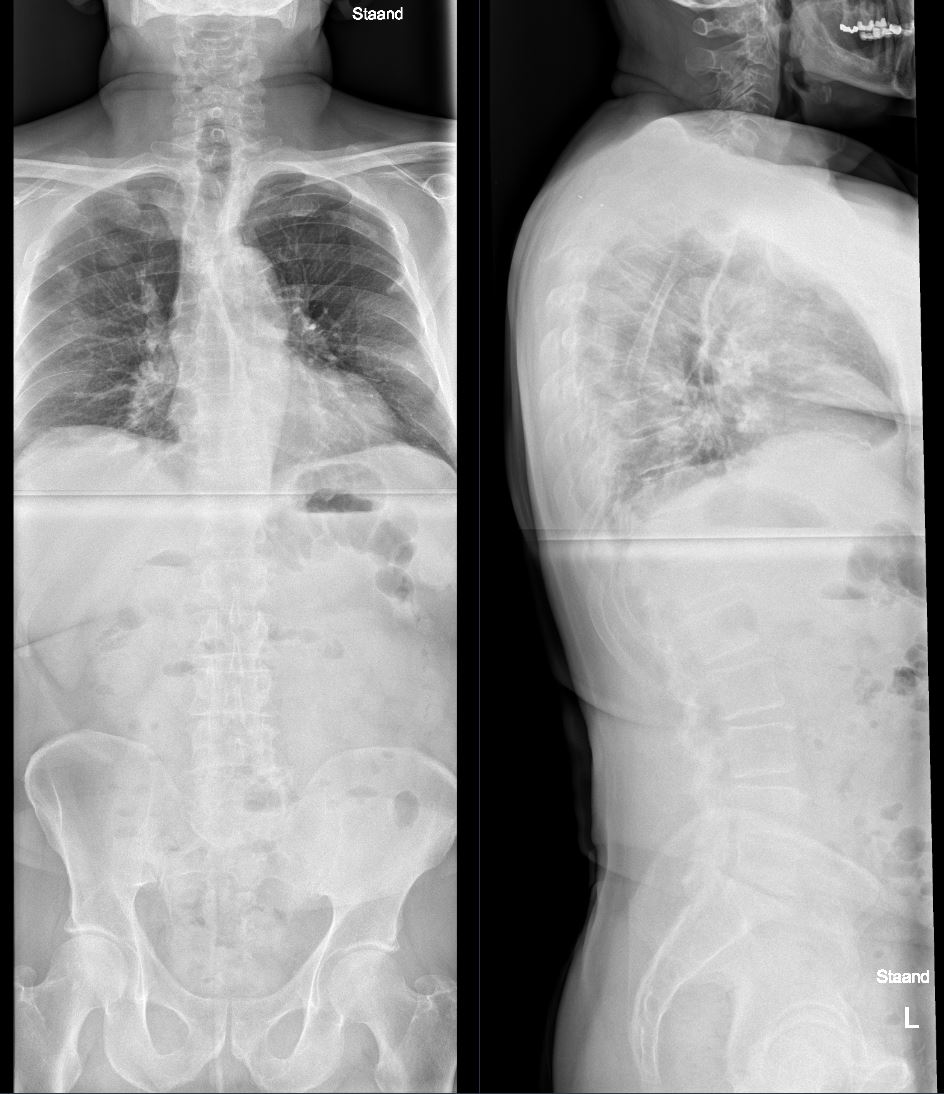 |
| --- |


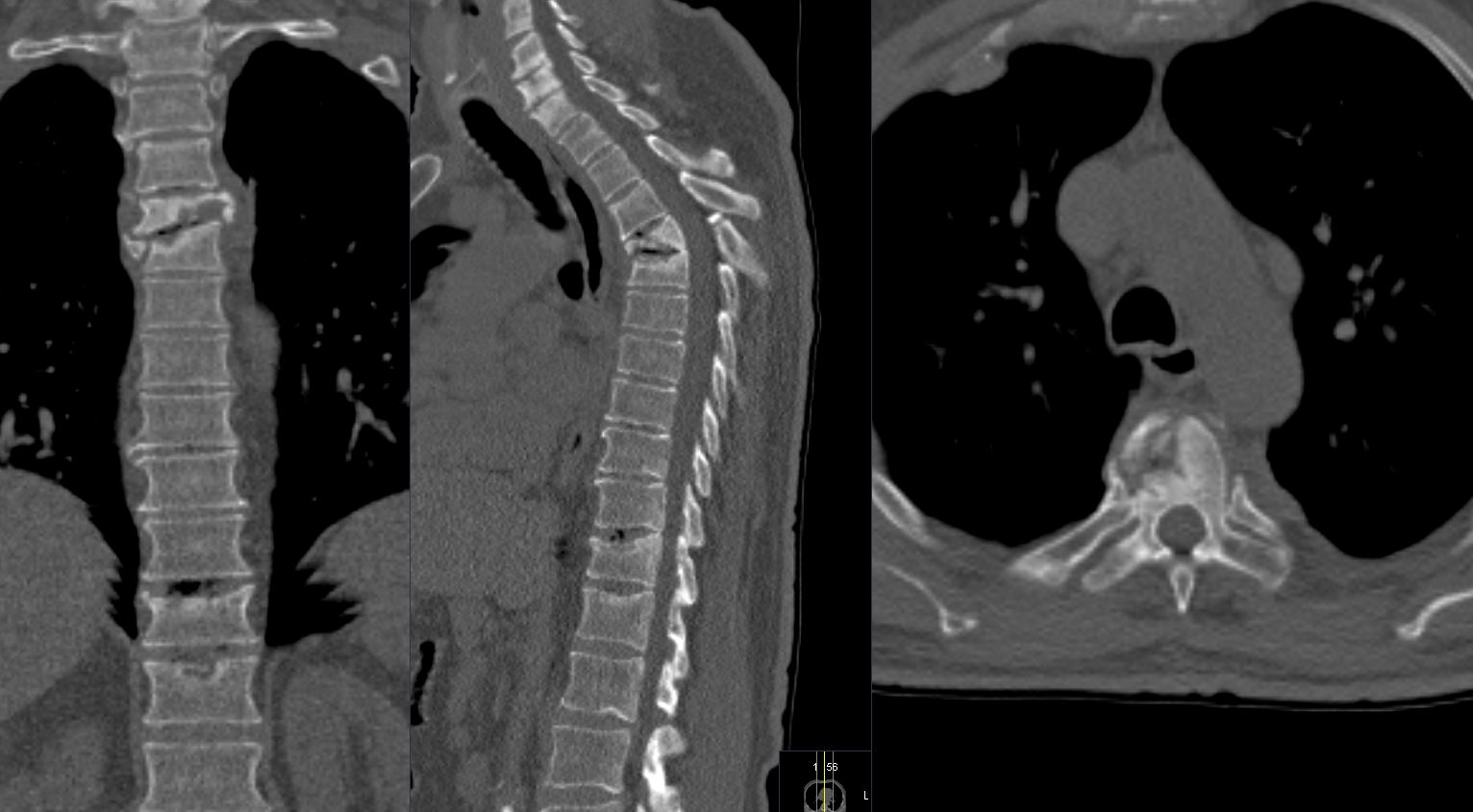

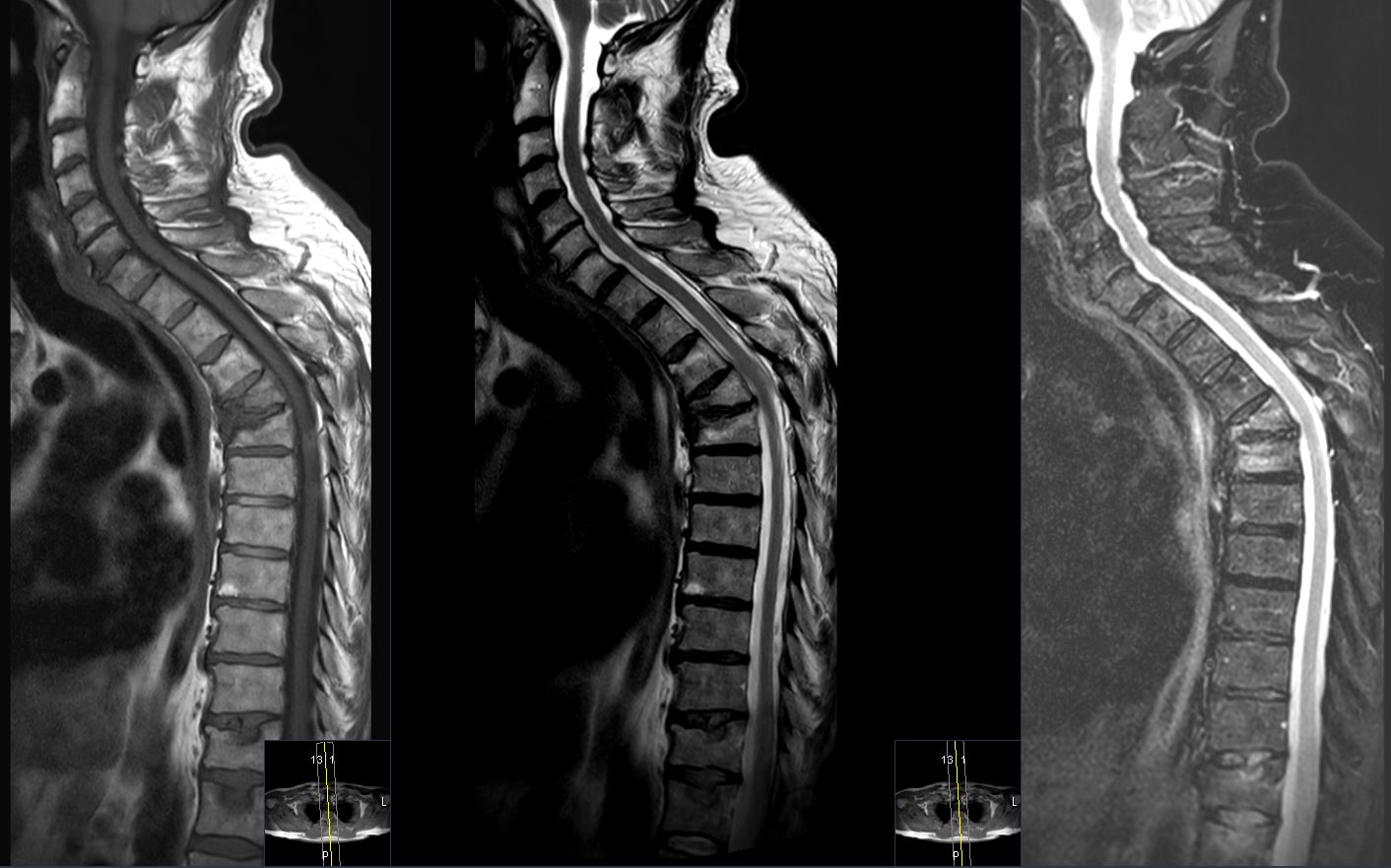


***Case 6***

Visit outpatient clinic

Male, age 63

Presentation 6 months after a fall, resulting in L2 A4, received operative treatment spondylodesis L1-3, still uses multiple painkillers

Current clinical presentation: Disabling progressive lower back pain 6 weeks after surgery, pain while walking

Physical examination: Painful palpation lumbar spine, all movement is painful, no neurological

Additional: Diabetes type 2, CABG, intermittent claudication. Work: retired (hospitality)

Radiological assessments and parameters

CT trauma: L2 A4 fracture, vertebral arch L2 is fractured

CT +6months: Pull out of superior right screw (L1), vacuum cleft L2.

Full spine radiograph +6months: Cobb(L1-L3): 14˚, wedge (L2): 25˚, ThK (T4-T12): 35˚, ThL(T11-L1) 6˚, LL (L1-L5): 24˚, SS: 23˚, PT: 24˚, PI: 46˚, SVA: 78mm


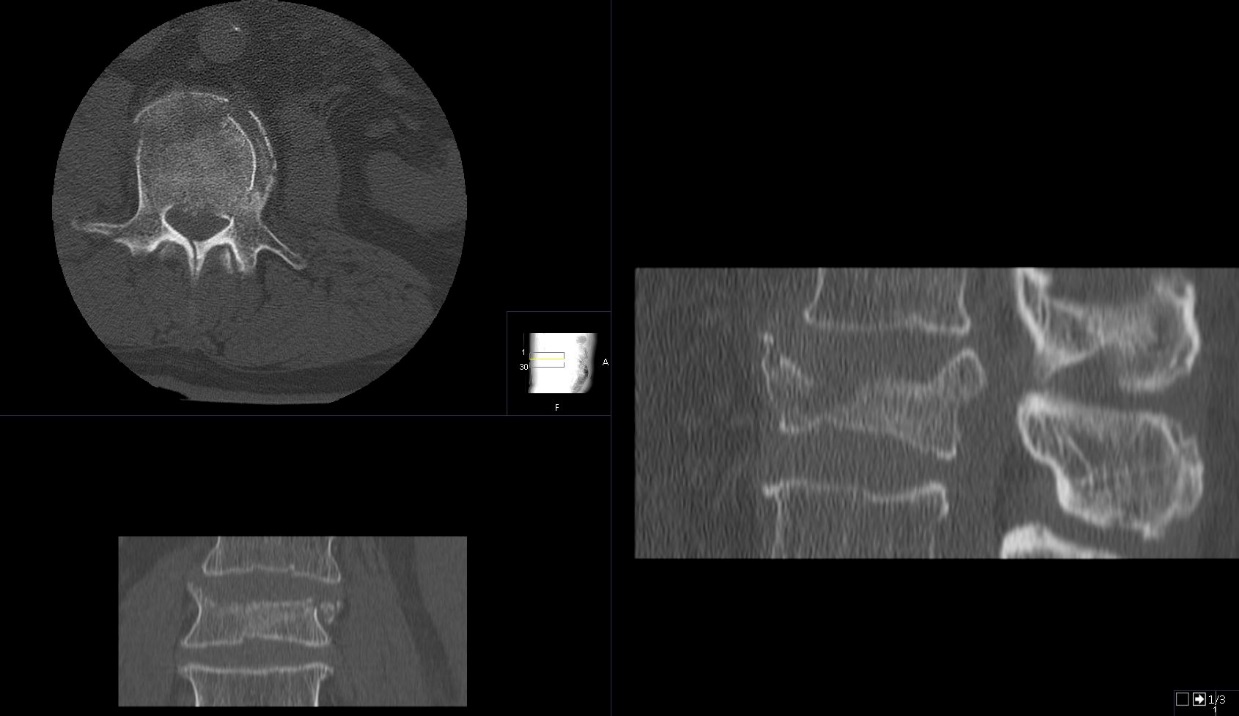

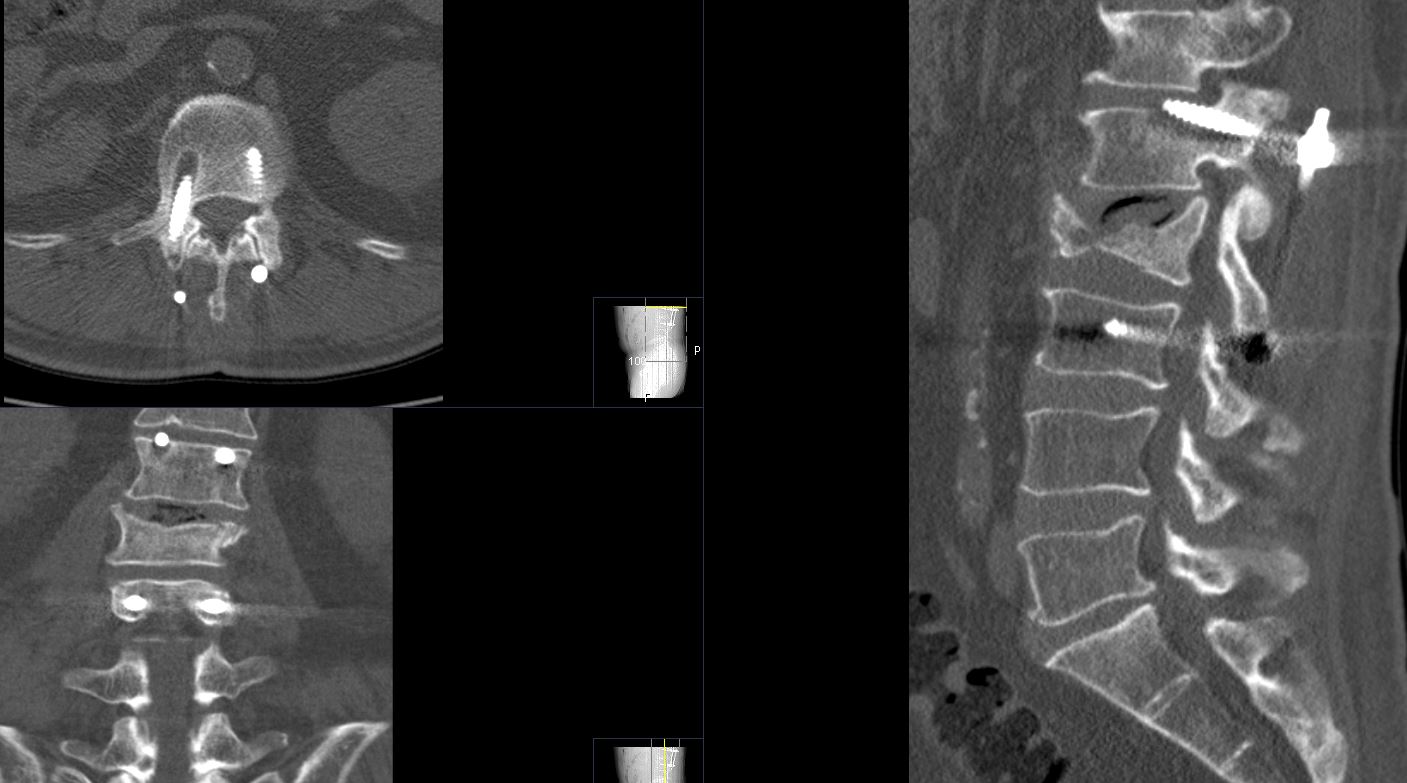


| 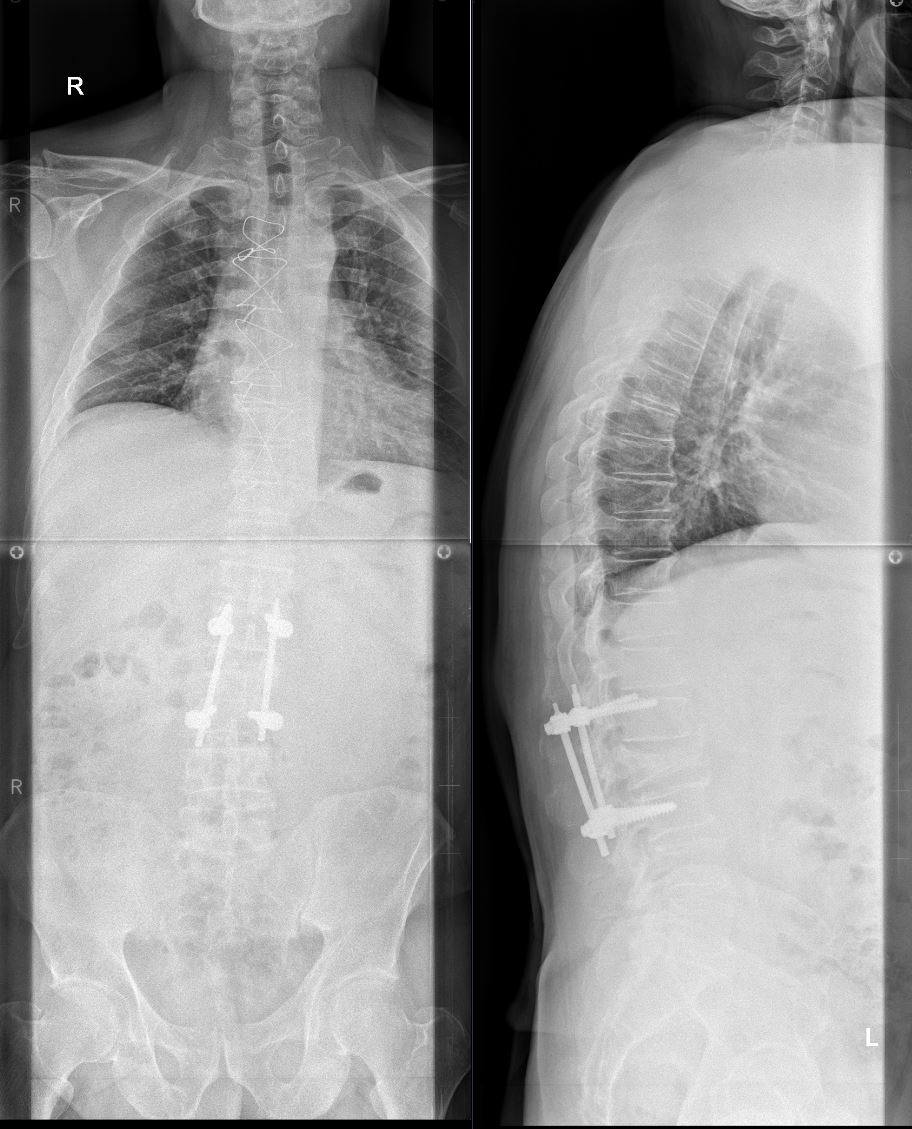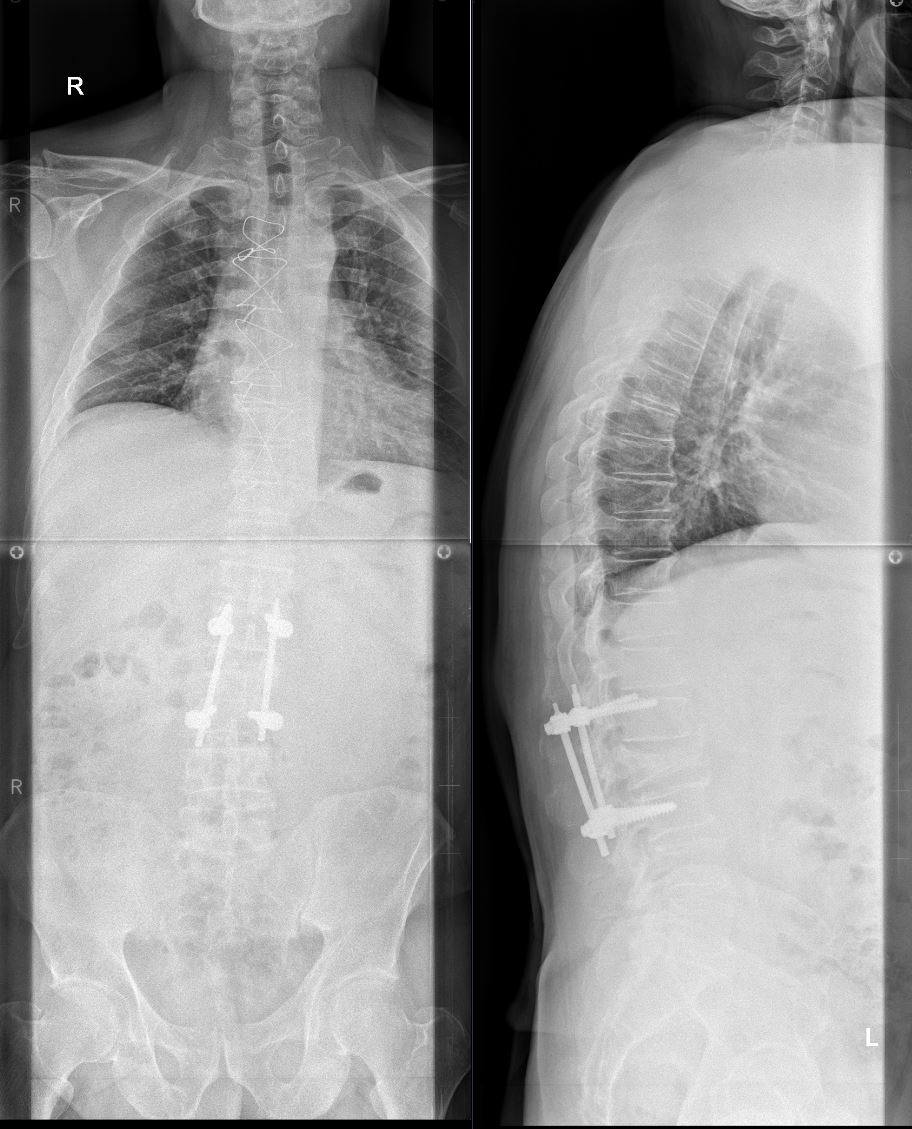 |
| --- |

***Case 7***

Visit outpatient clinic

Male, age 15

Presentation 5 months after someone hit his neck during PT-class, but CT-scan showed no traumatic injuries. Patient described a temporary funny feeling in both legs. Received conservative treatment of 1 week collar, and was discharged after 1 week from follow-up

Current clinical presentation: Persistent pain during the day, worse at night.

Physical examination: Extension, rotation and lateroflexion all partially restricted. No neurological deficit.

Additional: No medical history. Work: High school, aspires to work in construction

Radiological assessments and parameters

CT trauma: no traumatic abnormalities, slight kyphosis normal variant or due to pain

Cervical spine radiograph +5months: major kyphotic Cspine, Cobb (inf C2-C4): 53˚, wedge (C3): 13˚, C2-7 L: 17 ˚ kyphosis, C2-7 SVA: 21mm, T1 slope: 36˚

MRI +5months: Dislocation C2-C3, no myelopathy


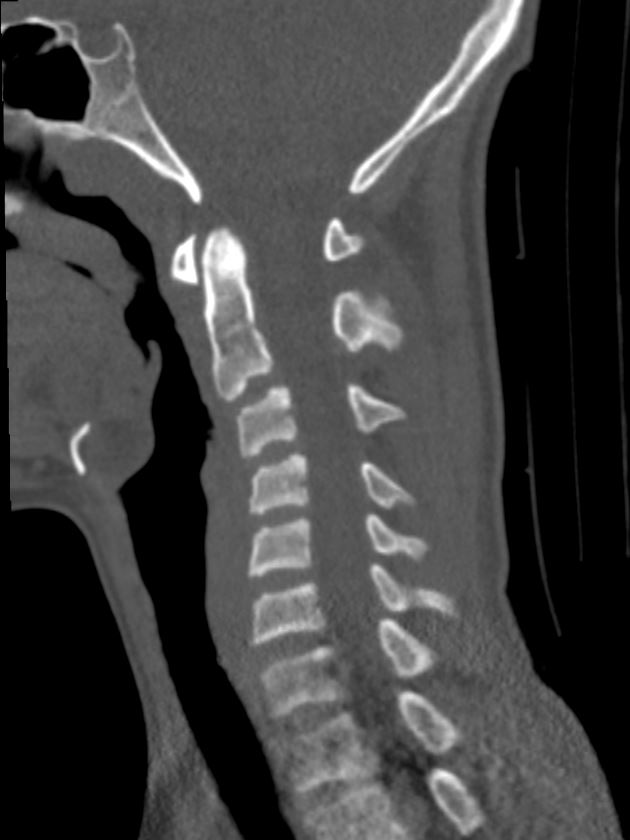


| 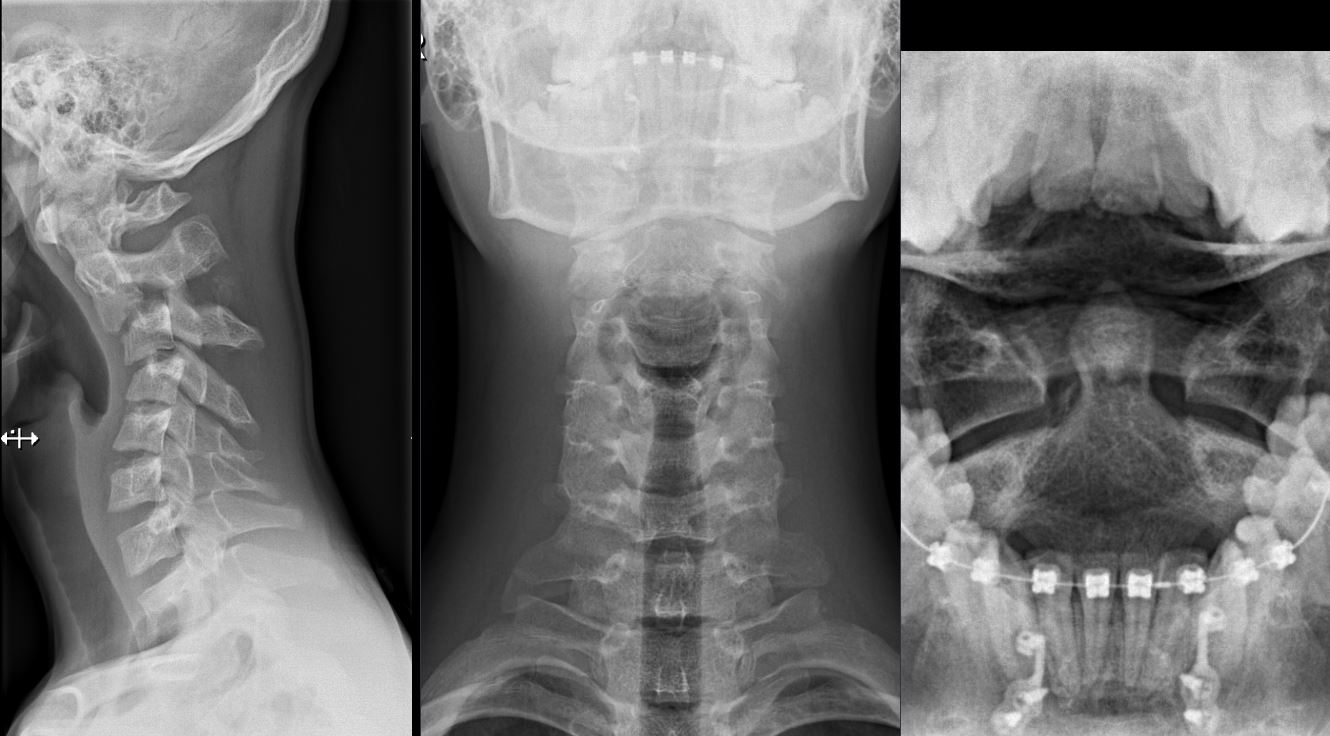 |
| --- |


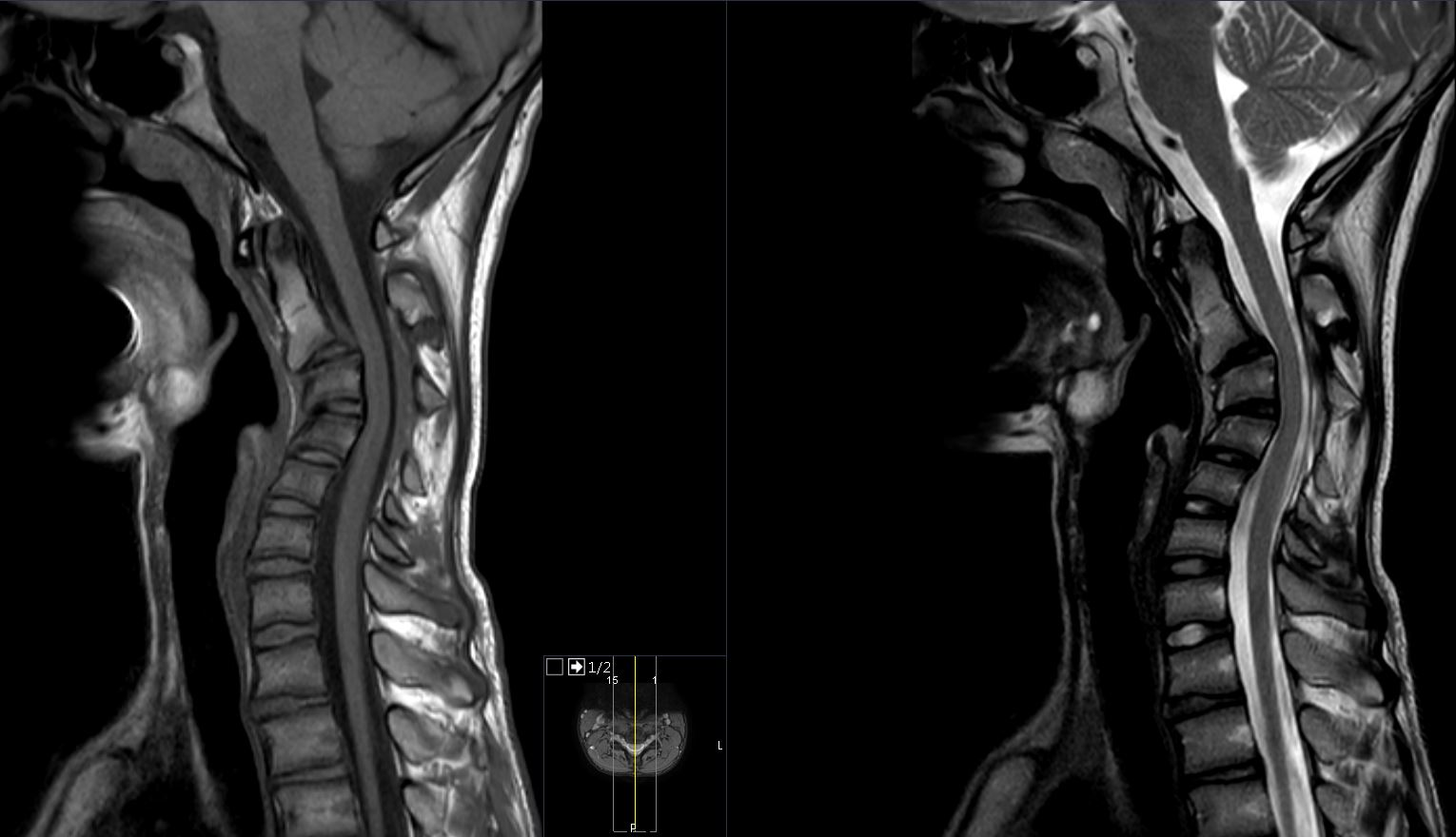

Supplement: Supplementary file 1 [file bsd-36-e383-s001.docx]
